# Genome-Wide Identification and Characterization of the *Wnt* Gene Family in Donkey (*Equus asinus*): Phylogenetic Analysis and Expression Profiling

**DOI:** 10.3390/biology15151323

**Published:** 2026-08-06

**Authors:** Qifei Zhu, Yadi Jing, Yunfan Zheng, Muhammad Zahoor Khan, Anqi Liu, Yongdong Peng, Changfa Wang

**Affiliations:** Liaocheng Research Institute of Donkey High-Efficiency Breeding and Ecological Feeding, College of Agriculture and Biology, Liaocheng University, Liaocheng 252000, Chinazahoorkhan@lcu.edu.cn (M.Z.K.);

**Keywords:** donkey, gene expression, genome, phylogenetic analysis, *Wnt* gene family

## Abstract

The *Wnt* gene family encodes highly conserved signaling molecules involved in vertebrate development, tissue homeostasis, and cell fate regulation. However, the evolutionary characteristics and potential biological functions of *Wnt* genes in the domestic donkey (*Equus asinus*) remain poorly understood. In this study, we performed the first genome-wide identification and comprehensive characterization of the donkey *Wnt* gene family. A total of 19 *Wnt* genes were identified, consistent with the conserved *Wnt* repertoire reported in other mammalian species. Phylogenetic and synteny analyses revealed strong evolutionary conservation of *Wnt* genes among mammals, particularly between donkey and horse, indicating conserved orthologous relationships within the genus *Equus*. Structural analyses showed that all donkey Wnt proteins contained the conserved cysteine-rich domain (CRD), whereas sequence variation was mainly concentrated in the N- and C-terminal regions. Transcriptomic analysis across 13 adult tissues revealed tissue-biased expression patterns, with *Wnt3*, *Wnt5a*, and *Wnt4* showing relatively higher expression in the testis, epididymis, and skin, respectively, suggesting their potential involvement in reproductive regulation and tissue homeostasis. PPI network and GO enrichment analyses indicated potential associations between donkey Wnt proteins and conserved regulators involved in developmental processes, cell fate regulation, and *Wnt* signaling pathways. Integration of expression profiles with predicted interaction networks further suggested tissue-associated regulatory patterns among *Wnt* pathway components. Overall, this study provides a comprehensive resource for understanding the genomic organization, evolutionary conservation, and expression diversity of the donkey *Wnt* gene family, and offers insights into potential Wnt-mediated regulatory mechanisms in donkey biology.

## Simple Summary

In this study, we systematically characterized the donkey *Wnt* gene family using genome-wide identification, phylogenetic analysis, gene structure and conserved motif analysis, chromosomal localization, and synteny comparison, revealing the genomic composition and evolutionary patterns of *Wnt* genes in donkeys. Furthermore, transcriptomic profiling across multiple tissues, Gene Ontology (GO) enrichment analysis, protein–protein interaction (PPI) network analysis, and subcellular localization prediction were integrated to explore the potential biological functions of donkey *Wnt* genes at the levels of tissue expression patterns and signaling regulation networks. This study provides molecular insights into the evolutionary conservation and functional diversification of the donkey *Wnt* gene family and offers theoretical foundations for donkey genetic resource conservation, regulation of economically important traits, and molecular breeding strategies.

## 1. Introduction

The *Wnt* gene family encodes a group of highly conserved, cysteine-rich secreted signaling glycoproteins that play pivotal regulatory roles in vertebrate embryonic development, tissue homeostasis, and cell fate determination in multicellular animals [1,2]. *Wnt* signaling mediates intercellular communication and transcriptional regulatory programs, thereby governing key biological processes such as body axis formation, organogenesis, tissue regeneration, and cell migration [3]. Thus, it constitutes a key regulatory network maintaining developmental integrity and tissue homeostasis in animals [4].

From an evolutionary perspective, the *Wnt* gene family is highly conserved across metazoans; however, gene duplication events and species divergence have led to progressive functional diversification and expression specialization, resulting in a characteristic evolutionary pattern of “structural conservation with functional diversification” [3,5]. Wnt proteins possess a conserved cysteine-rich domain (CRD) and undergo lipid modifications in the endoplasmic reticulum [6]. In addition to the CRD region, Wnt proteins contain multiple conserved sequence motifs that contribute to protein folding, structural stability, ligand secretion, and receptor recognition [7]. The evolutionary conservation of these motifs reflects functional constraints acting on essential regions, whereas motif divergence may indicate potential structural adaptation and functional specialization among *Wnt* members [8,9]. These structural features not only influence receptor binding affinity, secretion efficiency, and signaling range, but may also contribute to the functional specialization observed among different *Wnt* members [10]. Although Wnt proteins are generally considered canonical secreted signaling molecules, distinct members exhibit notable differences in subcellular trafficking, secretion dynamics, and tissue distribution, suggesting finely tuned regulatory control of their functional outputs [10,11].

Based on receptor engagement and downstream signaling mechanisms, *Wnt* pathways are generally divided into canonical β-catenin-dependent signaling and non-canonical β-catenin-independent pathways [12,13]. The canonical pathway primarily regulates transcriptional programs controlling cell proliferation, differentiation, and stem cell maintenance, whereas non-canonical pathways, including planar cell polarity (PCP) and Wnt/Ca^2+^ signaling, regulate cell polarity, migration, and tissue morphogenesis [14,15]. During vertebrate evolution, *Wnt* genes have been shaped by ancient genome duplication events and lineage-specific diversification [3], generating paralogous gene pairs such as *Wnt5a*/*Wnt5b* and *Wnt7a*/*Wnt7b* with distinct expression patterns and biological functions [16,17].

Recent comparative genomic studies in domestic animals have demonstrated that *Wnt* gene families are highly conserved while undergoing lineage-specific regulatory diversification [18]. In cattle, genome-wide analyses identified 19 *Wnt* genes with conserved exon–intron structures, protein motifs, and phylogenetic relationships, whereas transcriptomic analyses revealed distinct tissue-specific expression patterns, suggesting that *Wnt* members retain conserved developmental functions while acquiring species-specific regulatory features [19]. Similar evolutionary patterns have been observed in other livestock species, highlighting the essential roles of *Wnt* signaling in vertebrate development and physiological regulation [20]. For example, in sheep, activation of the Wnt/β-catenin pathway promotes hair follicle development and wool production by regulating downstream keratin and keratin-associated protein genes [21,22]. In pigs and rodents, *Wnt* signaling components have been implicated in adipogenesis, skeletal development, stem cell maintenance, and tissue regeneration through the regulation of cell fate determination and progenitor cell activity [23,24,25,26]. However, comprehensive genome-wide characterization of *Wnt* gene families, integrating gene structure, evolutionary relationships, and tissue expression patterns, remains limited in many non-model and economically important vertebrate species.

The domestic donkey (*Equus asinus*) is an important livestock species with agricultural, nutritional, and adaptive significance [27]. Long-term domestication has shaped distinctive traits, including tolerance to harsh environments, efficient feed utilization, and stable physiological performance [28,29,30,31]. In particular, donkey milk has received increasing attention due to its nutritional properties and similarity to human milk composition, expanding its potential applications in functional food and specialized nutrition [32,33]. Therefore, understanding the molecular networks regulating growth, reproduction, and tissue function is important for improving donkey genetic resources and breeding strategies [30].

Despite advances in donkey genome resources, systematic analyses of key developmental regulatory gene families remain limited [34]. To date, the genomic organization, evolutionary relationships, structural characteristics, and tissue-specific expression patterns of the *Wnt* gene family in donkeys have not been comprehensively investigated. Moreover, the extent to which donkey *Wnt* genes maintain evolutionary conservation or exhibit functional diversification remains unclear. Given the fundamental roles of *Wnt* signaling in vertebrate development, tissue homeostasis, and regeneration, systematic characterization of the donkey *Wnt* family may provide valuable insights into the molecular mechanisms underlying important biological traits and genetic improvement of this species [2,35].

To address this knowledge gap, this study performed the first systematic genome-wide investigation of the *Wnt* gene family in *Equus asinus*. By integrating phylogenetic analysis, gene structure characterization, conserved motif identification, chromosomal distribution, synteny analysis, protein structural prediction, comparative expression profiling, and functional network analysis, we provide a comprehensive overview of the evolutionary conservation and potential functional diversification of donkey *Wnt* genes. Furthermore, tissue-specific expression profiles and cross-species expression analyses were used to explore the potential functions of *Wnt* signaling components in donkeys. Functional network analysis further revealed their possible regulatory roles. These findings provide new insights into the conserved and divergent functions of *Wnt* signaling components in donkey development, reproduction, and tissue homeostasis. Collectively, this study establishes the first systematic genomic framework for the *Wnt* signaling system in donkeys and provides valuable molecular resources for further investigating Wnt-mediated biological processes in *Equus asinus*.

## 2. Materials and Methods

### 2.1. Genome-Wide Identification of Wnt Genes

**Figure 1:**
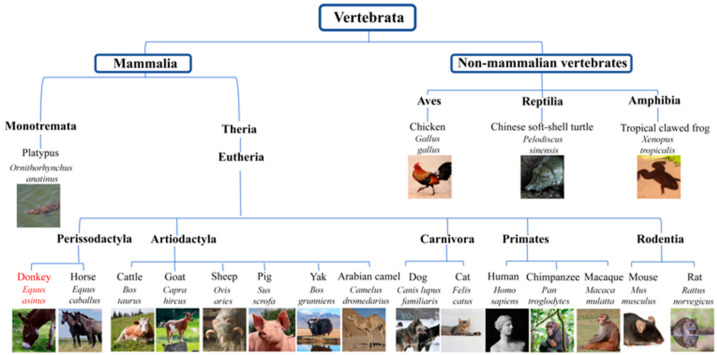
Species used for comparative genomic analysis of the *Wnt* gene family. The species highlighted in red represents the target organism investigated in this study (*Equus asinus*, donkey).

General feature format version 3 (GFF3) annotation files, genome sequences, and protein sequence data for 19 eukaryotic organisms (Figure 1) were downloaded from the ENSEMBL database [36]. The analyzed species included donkey (*Equus asinus*), yak (*Bos grunniens*), Arabian camel (*Camelus dromedarius*), tropical clawed frog (*Xenopus tropicalis*), Chinese soft-shell turtle (*Pelodiscus sinensis*), chicken (*Gallus gallus*), platypus (*Ornithorhynchus anatinus*), cat (*Felis catus*), dog (*Canis lupus familiaris*), horse (*Equus caballus*), pig (*Sus scrofa*), cattle (*Bos taurus*), goat (*Capra hircus*), sheep (*Ovis aries*), mouse (*Mus musculus*), rat (*Rattus norvegicus*), macaque (*Macaca mulatta*), chimpanzee (*Pan troglodytes*), and human (*Homo sapiens*). These species were selected to represent major vertebrate lineages, including closely related equids, diverse mammals, and non-mammalian vertebrates, enabling a comparative analysis of *Wnt* gene conservation and evolutionary diversification. To enhance the rigor of *Wnt* gene family identification, conserved domain validation was further conducted using the Pfam database [37]. All candidate Wnt protein sequences were scanned against the Pfam-A database with HMMER (v3.4) [38], employing the canonical Wnt-family hidden Markov model PF00110. Protein sequences harboring a complete PF00110 domain were considered putative *Wnt* members. This Pfam-based screening step was integrated with genome annotation information, HMMER-based profile searches, and subsequent conserved domain confirmation using both CDD [39] and SMART [40], thereby ensuring high-confidence and reliable identification of *Wnt* family members in donkeys.

### 2.2. Analysis of Wnt Protein Characteristics

The physicochemical properties of donkey Wnt proteins were evaluated using the ExPASy ProtParam tool [41]. Sequence characteristics, including amino acid length, theoretical isoelectric point (pI), and molecular weight (MW), as well as stability- and hydrophobicity-related parameters, including instability index, aliphatic index, and GRAVY, were analyzed. Subcellular localization was predicted using DeepLoc [42]. Signal peptide prediction was performed using SignalP 6.0 [43], and proteins with a signal peptide probability score (SP_score) ≥ 0.5 were considered to contain potential signal peptides. Transmembrane helices were predicted using TMHMM [44]. Secondary structure composition, including α-helices, β-sheets, turns, and random coils, was predicted using the SOPMA server [45]. Three-dimensional structures of donkey Wnt proteins were obtained from the AlphaFold Protein Structure Database [46]. The predicted structures were evaluated based on the per-residue confidence score (pLDDT), which was used to assess the reliability of structural predictions [47].

### 2.3. Phylogenetic Analysis

Donkey Wnt protein sequences were retrieved from the ENSEMBL database. Phylogenetic relationships among *Wnt* gene family members were inferred using the maximum likelihood (ML) method implemented in MEGA12 [48]. Bootstrap support was estimated with 1000 replicates, and only nodes with bootstrap values ≥ 70% are reported. Bayesian inference (BI) analysis was performed using MrBayes, and an additional BI analysis based on the same dataset with 1000 replicates is provided in the Supplementary Materials to further validate the robustness of the phylogenetic results [49]. For the species-specific phylogenetic analysis presented in Supplementary Figure S4, full-length amino acid sequences of donkey and horse Wnt proteins were used to construct a separate ML tree using the same parameters described above. The resulting phylogenetic trees were visualized and annotated using Interactive Tree Of Life (iTOL) [50]. To further explore global similarity patterns among *Wnt* genes across species, multidimensional scaling (MDS) analysis was conducted in R using the cmdscale function from the stats package based on pairwise amino acid sequence distances [51].

### 2.4. Chromosomal Localization and Gene Structure Analysis

The chromosomal localization of donkey *Wnt* genes was determined using the “GeneLocation Visualize” function in TBtools software (version 2.441). Gene structure visualization was performed using the “VisualizeGeneStructure” plugin in TBtools [52]. Synteny and collinearity analyses between donkey (*Equus asinus*) and horse (*Equus caballus*) genomes were performed using the MCScanX toolkit, and *Wnt* gene syntenic relationships were visualized based on the generated synteny plots [53].

### 2.5. Conserved Motif Analysis

Conserved protein motifs within the donkey *Wnt* gene family were identified using the MEME Suite online tool [54]. The maximum number of motifs was set to 10, with other parameters maintained at the default settings. In addition, Wnt protein sequences were analyzed using the NCBI Batch CD-Search tool [55] to identify conserved domains. The motif and conserved domain information obtained from the MEME and CD-Search analyses were integrated and visualized using the “GeneStructureView” plugin in TBtools software. The conservation level of each identified motif was evaluated based on the mean conservation score of amino acid sites within the motif. Conservation scores of individual amino acid positions were calculated using an in-house R script based on amino acid residue frequencies derived from multiple sequence alignment. Motifs with relatively higher mean conservation values (>0.7) were considered highly conserved in this study [56].

### 2.6. Protein–Protein Interaction (PPI) Network Analysis

Protein–protein interaction (PPI) networks of donkey Wnt proteins were predicted using the STRING web interface [57], with *Equus asinus* selected as the reference species. Only high-confidence interactions (interaction score ≥ 0.9) were included, and the resulting network was exported and visualized using Cytoscape (v3.10.3) [58].

### 2.7. Tissue Expression Analysis

Adult donkey tissue RNA-Seq datasets were obtained from the NCBI SRA database (BioProject: PRJNA431818) [59]. The tissue expression patterns of donkey *Wnt* family genes were analyzed using transcriptomic data from 13 adult tissues, including heart, kidney, liver, lung, brain, spleen, stomach, blood, skin, cecum, muscle, testis, and epididymis. For each tissue, three independent biological replicates from different individuals were included for gene expression analysis. Raw sequencing reads were subjected to quality assessment utilizing FastQC software (v0.11.8), and clean reads were aligned based on the donkey reference genome assembly ASM1607732v2 using HISAT2 software (v2.1.0) [29,60]. Gene expression levels were quantified using StringTie (v1.0.4) and expressed as fragments per kilobase of exon per million mapped reads (FPKM) [61]. To visualize *Wnt* family gene expression profiles across tissues, the FPKM expression matrix was transformed using log2(FPKM + 1) and used for heatmap construction. Hierarchical clustering was performed based on Euclidean distance and complete linkage method to group *Wnt* genes and tissues according to their expression similarity patterns. The color scale represents the relative expression abundance after log2 transformation, with the unit indicated as log2(FPKM + 1). Genes with FPKM values ≥ 1 were considered detectable, whereas genes below this threshold were regarded as undetectable or lowly expressed. Tissue expression preferences of *Wnt* family genes were evaluated based on relative expression abundance among different tissues using the normalized expression matrix. To provide cross-species comparative context, representative *Wnt* genes were selected based on tissue expression patterns identified in donkey and their tissue expression profiles in horse and cattle were retrieved from the Bgee database [62]. The top 15 tissues ranked by expression score were selected for comparative analysis. Expression scores and false discovery rate (FDR) values were used to evaluate tissue-associated expression patterns.

### 2.8. Functional Enrichment Analysis

The functional characterization of the donkey *Wnt* gene family was performed using Gene Ontology (GO) enrichment analysis based on Biological Process (BP), Molecular Function (MF), and Cellular Component (CC) categories. GO enrichment analysis was conducted using the DAVID online platform [63].

### 2.9. Data Processing and Statistical Analysis

Normalized FPKM values were used to evaluate the expression patterns of *Wnt* family genes across different donkey tissues. Tissue-preferential expression patterns were evaluated based on normalized expression abundance and tissue enrichment profiles. Hierarchical clustering and heatmap visualization were performed to compare *Wnt* gene expression patterns among tissues, with interpretations focused on tissue distribution rather than differential expression analysis.

For Gene Ontology (GO) enrichment analysis, significantly enriched terms were identified using Benjamini–Hochberg corrected *p*-values. Terms with adjusted *p*-value < 0.05 were considered statistically significant.

## 3. Results

### 3.1. Identification and Characterization of Donkey Wnt Gene Family Members

Using the HMMER (version 3.4) and BLAST (version 2.17.0) algorithms, we systematically searched the genome and proteome databases to identify putative *Wnt* family members, with subsequent validation of complete *Wnt* domains via the Conserved Domain Database (CDD). This comprehensive approach resulted in the identification of 19 *Wnt* gene family members in the donkey genome (Table 1).

Sequence analysis revealed considerable variation in protein length among donkey Wnt family members, ranging from 349 amino acids (Wnt8b) to 557 amino acids (Wnt5b). The predicted molecular masses of these proteins ranged from 38.74 kDa to 62.56 kDa (Table 2). The instability index ranged from 35.80 (Wnt7a) to 62.81 (Wnt10a), whereas the aliphatic index values ranged from 59.81 (Wnt3a) to 72.35 (Wnt9b). Notably, all Wnt family members presented negative grand average of hydropathicity (GRAVY) values, indicating their hydrophilic characteristics.

Secondary structure prediction using SOPMA indicated that donkey Wnt proteins were predominantly composed of random coils, followed by α-helices, whereas extended strands and β-turns represented the least abundant components (Table 3). The proportions of α-helices, extended strands, β-turns, and random coils varied among Wnt members, ranging from 25.84–40.00%, 8.15–12.16%, 1.67–4.67%, and 47.32–63.51%, respectively. Integration of AlphaFold models with the predicted Local Distance Difference Test (pLDDT) scores revealed domain-specific structural features of Wnt proteins (Figure S1) [46,47]. The cysteine-rich domain (CRD) exhibited relatively higher confidence scores and more ordered structural characteristics, whereas the N- and C-terminal regions showed lower confidence values and predominantly disordered conformations. β-Structural elements were sparsely distributed across *Wnt* sequences. These structural patterns were consistent with the SOPMA predictions, indicating a conserved secondary structural framework among donkey Wnt proteins. Further analysis revealed that proline abundance was higher in Wnt proteins with a lower α-helix content (Table S1), consistent with previous studies describing the influence of proline on protein secondary structure [64,65,66]. Correlation analysis showed that α-helix proportion was negatively correlated with loop proportion and positively correlated with pLDDT scores, whereas β-sheet-related elements showed no significant correlations with other secondary structure components (Table S2).

Subcellular localization prediction indicated that all 19 donkey Wnt proteins were most likely localized to the extracellular region, with predicted probabilities ranging from 0.69 to 0.91. Further analysis of signal peptides and transmembrane helices revealed that seven Wnt proteins were predicted to contain signal peptides, including Wnt1, Wnt10a, Wnt10b, Wnt2, Wnt3, Wnt3a, and Wnt9b. Among these proteins, five members were additionally predicted to contain transmembrane helices. In contrast, Wnt4, Wnt5a, and Wnt16 exhibited relatively high predicted extracellular localization probabilities but lacked obvious signal peptide features, suggesting that conventional signal peptide prediction approaches may not fully capture the diverse secretion mechanisms of Wnt proteins. Cross-species normalized comparisons across four placental mammals (human, cattle, horse, and donkey) further supported this pattern, with extracellular localization consistently ranked as the dominant prediction among all subcellular compartments (Figure S2).

### 3.2. Structural Architecture of the Donkey Wnt Gene Family

**Figure 2:**
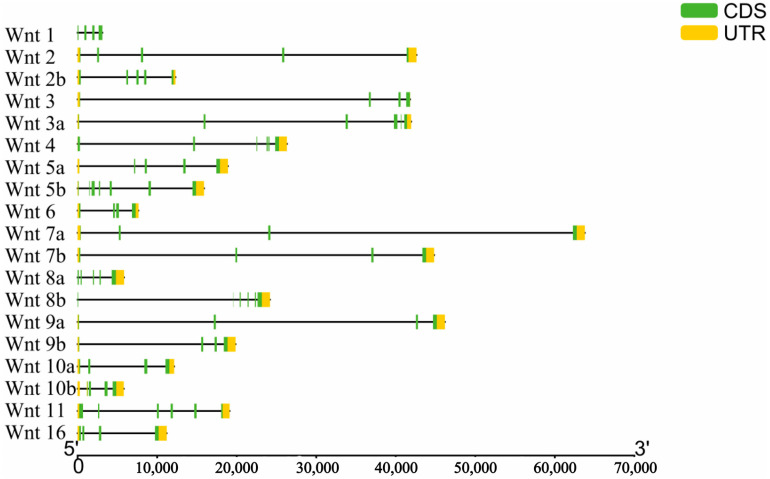
Structural analysis of *Wnt* gene family members in donkeys. Yellow boxes indicate coding sequences (UTRs), green boxes represent untranslated regions (CDSs), and black lines denote introns.

Genome annotation analysis revealed that donkey *Wnt* genes were unevenly distributed across 12 chromosomes, with exon numbers ranging from 4 to 7 (Figure 2). *Wnt3*, *Wnt6*, *Wnt7a*, *Wnt7b*, *Wnt9a*, and *Wnt16* contained four exons, whereas *Wnt5b* and *Wnt11* contained seven exons. The remaining *Wnt* genes generally consisted of five to six exons.

**Figure 3:**
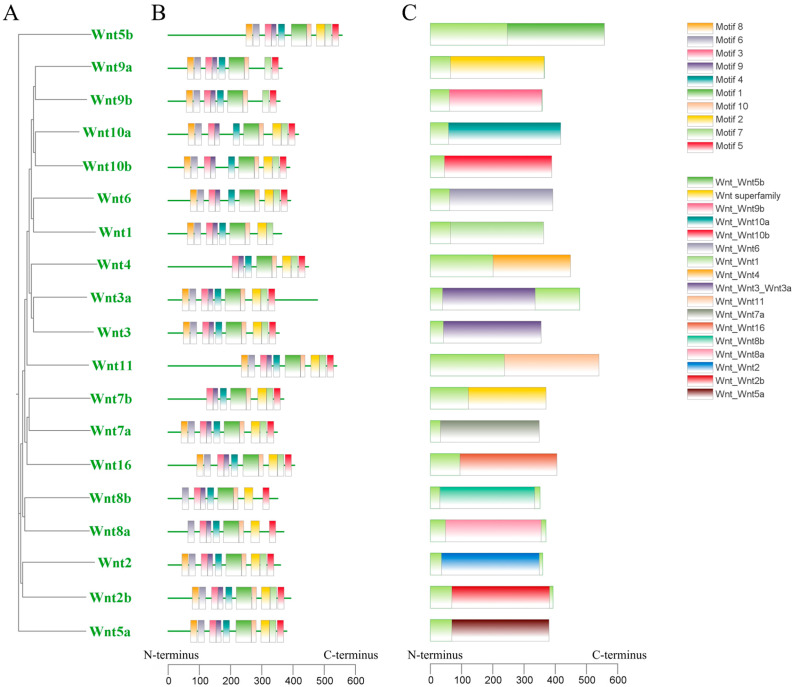
Phylogenetic relationships, conserved protein motifs, and conserved domains of donkey Wnt proteins. (**A**) Phylogenetic relationships of donkey Wnt proteins inferred using the maximum likelihood method. (**B**) Distribution of conserved motifs identified by MEME. Each colored box represents an individual conserved motif, and different colors correspond to different motif numbers (Motif 1–Motif 10) as indicated in the legend. The X-axis indicates amino acid coordinates within each Wnt protein. (**C**) Conserved domains identified using the NCBI Batch CD-Search tool. Colored bars represent different conserved domain types, and the corresponding domain names are shown in the legend. The X-axis indicates amino acid coordinates within each Wnt protein.

Conserved motif analysis revealed a highly conserved motif architecture among donkey Wnt proteins, with all members retaining motifs 1, 3, 4, and 9, and 12 proteins containing the complete set of 10 predicted motifs (Figure 3). Motif conservation analysis showed differential sequence constraints among motifs (Table S4). Motifs 1, 5, 6, and 9 exhibited relatively high conservation (mean conservation > 0.70), with motif 9 being completely conserved across all sequences, consistent with the evolutionary conservation of functionally constrained regions in Wnt proteins [7,8,9]. In contrast, motifs 4, 8, and 10 displayed lower conservation levels, indicating greater sequence variability. Motif composition and conservation patterns were largely consistent within *Wnt* subfamilies (e.g., *Wnt3*/*Wnt3a*) and paralogous pairs (e.g., *Wnt9a*/*Wnt9b*).

To evaluate the primary sequence conservation of donkey Wnt proteins, multiple sequence alignment of the 19 Wnt amino acid sequences was performed (Supplementary Material File). The alignment revealed a highly conserved cysteine-rich core region among all *Wnt* members, consistent with the characteristic structural features of Wnt proteins. In contrast, sequence divergence was mainly observed in the N-terminal and C-terminal regions, which showed relatively lower conservation among *Wnt* members.

### 3.3. Phylogenetic Relationships of the Wnt Gene Family

**Figure 4:**
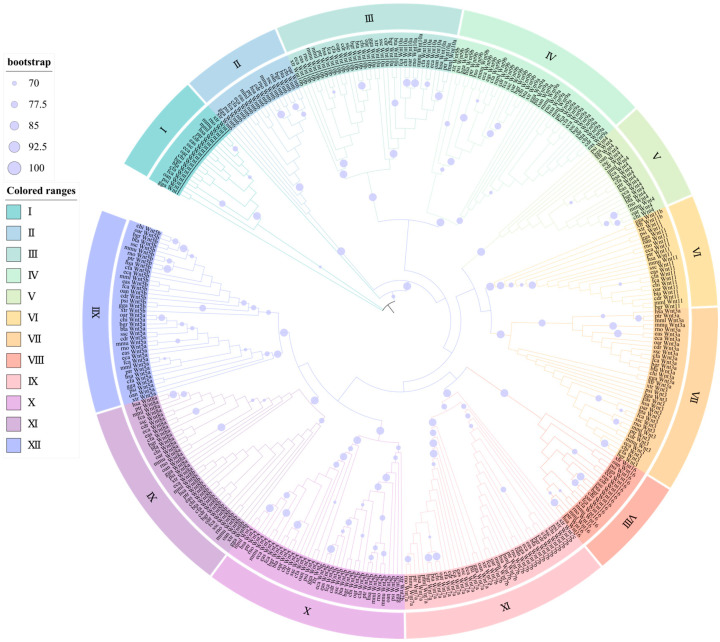
Phylogenetic maximum likelihood (ML) tree of *Wnt* proteins from 19 organisms. The purple circles at each branch node represent bootstrap support values, with circle size proportional to bootstrap confidence ranging from 70 to 100.

Phylogenetic analysis of *Wnt* genes across 19 vertebrate species revealed clear subfamily-specific clustering and species-related grouping patterns, reflecting the conserved evolutionary history of the *Wnt* gene family (Figure 4). Orthologous genes from mammals generally clustered together, and species-level groupings were often supported by moderate to high bootstrap values, although several deeper internal nodes exhibited relatively weak support. The equid orthologs of *Wnt8a* formed a strongly supported clade (bootstrap = 95), indicating close evolutionary relationships. Highly conserved members, including *Wnt1* and *Wnt10a*, displayed extremely short branch lengths across mammalian taxa, consistent with strong purifying selection. In contrast, *Wnt5a*, and particularly *Wnt7b*, showed evidence of lineage-specific divergence, with several taxa exhibiting noticeably elongated branches. Early-diverging vertebrates, including amphibian, avian, and basal vertebrate representatives, formed distinct clades with variable support values. Notably, *Xenopus laevis* sequences frequently exhibited longer branch lengths than their mammalian counterparts, suggesting accelerated evolutionary rates in this lineage. The high concordance between the ML and BI phylogenies further supports the robustness and reliability of these inferred evolutionary relationships (Figure S3).

To explicitly validate the annotation of donkey *Wnt* genes against closely related equids, a maximum-likelihood phylogenetic tree was constructed using only horse (*Equus caballus*) and donkey (*Equus asinus*) sequences (Figure S4). In this tree, all donkey *Wnt* genes clustered as sisters to their horse counterparts, with bootstrap support ≥ 0.7 for all nodes, confirming one-to-one orthologous relationships. This targeted analysis provides additional phylogenetic support for the reliability of donkey *Wnt* gene identification and demonstrates evolutionary consistency between donkey and horse *Wnt* gene repertoires.

**Figure 5:**
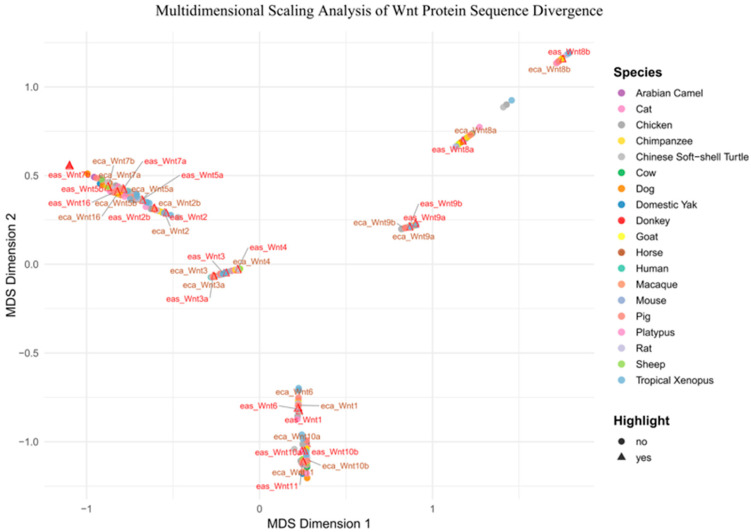
Multidimensional scaling (MDS) analysis based on pairwise amino acid sequence distances of Wnt proteins across vertebrate species. Each point represents an individual Wnt protein, and the analysis was performed using a pairwise amino acid sequence distance matrix. Points are colored according to species, while donkey Wnt proteins are highlighted using enlarged red triangular markers. The x- and y-axes represent dimensionless coordinates generated by MDS transformation of the sequence distance matrix. The relative positions of points reflect overall sequence similarity relationships, with closer points indicating greater similarity among Wnt protein sequences.

To complement the topology-based phylogenetic inference, multidimensional scaling (MDS) analysis was employed to visualize overall sequence similarity patterns among Wnt proteins across species (Figure 5). In the MDS space, donkey Wnt protein sequences clustered with sequences from evolutionarily closer mammalian species, such as horse and cattle, whereas more distantly related taxa, including Xenopus and chicken, occupied clearly separated positions. Unlike phylogenetic trees, which emphasize branching order and ancestry, MDS highlights global distance relationships shaped by sequence conservation and functional constraints. The concordance between MDS spatial partitioning and phylogenetic clustering indicates that donkey Wnt proteins not only follow established vertebrate evolutionary relationships, but also retain conserved sequence features within subfamilies, underscoring the biological relevance of MDS as a complementary approach to phylogenetic analysis.

### 3.4. Chromosomal Distribution and Synteny Analysis

**Figure 6:**
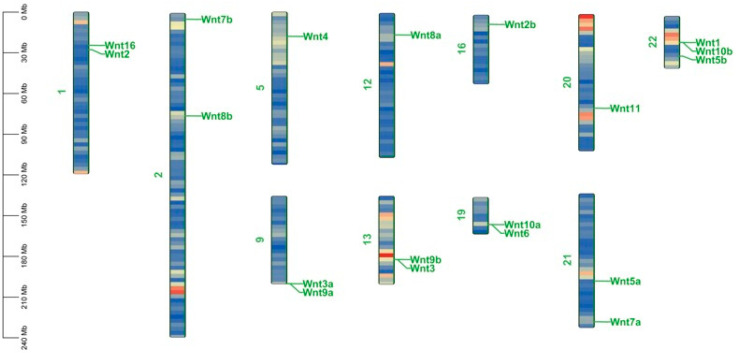
Chromosome localization of the donkey *Wnt* gene family. Numerals represent chromosome numbers; the vertical axis denotes chromosomal physical position (Mb). Chromosomal color gradients indicate genomic gene density, with red areas corresponding to high-density regions. Green labels mark the locations of individual *Wnt* genes.

Chromosomal localization analysis based on the donkey genome annotation revealed that the 19 *Wnt* genes were distributed across multiple chromosomes and exhibited a dispersed distribution pattern without extensive genomic clustering. Most chromosomes harbored one or more *Wnt* members. Notably, two closely linked gene pairs were identified: *Wnt3a* and *Wnt9a* on chromosome 9, and *Wnt3* and *Wnt9b* on chromosome 13 (Figure 6).

**Figure 7:**
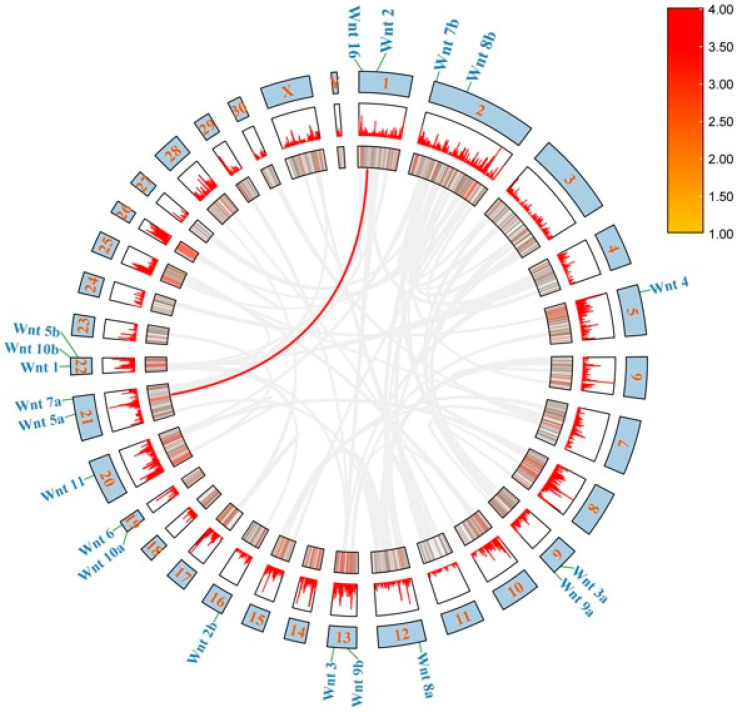
Circos plot showing the genomic distribution and syntenic relationships of donkey *Wnt* genes. The outermost blue segments represent donkey chromosomes, and blue labels indicate the chromosomal positions of *Wnt* genes. The red histogram track represents the number and distribution of *Wnt* genes across genomic regions, while the inner track displays *Wnt* gene density calculated within genomic windows. The color gradient scale represents relative gene density values, with yellow to red indicating increasing density values (1–4). Internal links indicate syntenic relationships among *Wnt* genes.

To explore the genomic organization of the *Wnt* gene family, a Circos plot integrating chromosomal positions and synteny relationships was constructed (Figure 7). The results revealed a largely conserved genomic distribution of *Wnt* genes in the donkey genome. Only a single collinearity signal involving the genomic regions harboring *Wnt2* and *Wnt5a* was detected; however, these genes belong to distinct and evolutionarily ancient *Wnt* subfamilies and do not represent a recent duplication-derived paralogous pair. No additional syntenic relationships indicative of lineage-specific or recent gene duplication were observed within the *Wnt* gene family in donkeys.

**Figure 8:**
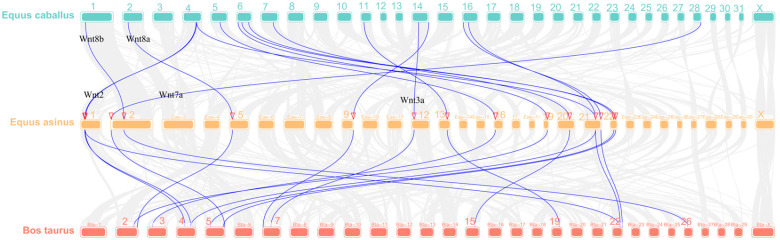
Collinearity analysis of the *Wnt* gene among donkeys, horses, and cattle. Blue curves indicate collinear orthologous Wnt gene pairs between species, whereas grey lines represent all genomic collinear blocks. Different colors represent different species: green for *Equus caballus*, orange for *Equus asinus*, and red for *Bos taurus*. Numbers above each chromosome represent chromosome identifiers, and gene labels indicate the corresponding Wnt gene positions within each species.

Comparative synteny analysis among donkey (2*n* = 62), horse (2*n* = 64), and cattle (2*n* = 60) genomes revealed a high degree of conservation of *Wnt* gene loci across species (Figure 8). Most *Wnt* genes exhibited conserved chromosomal positions among the three species, with several loci showing positional variation, including *Wnt3a*, *Wnt2*, *Wnt7a*, *Wnt8a*, and *Wnt8b*. These genes still retained identifiable orthologous syntenic relationships despite differences in genomic location. Overall, *Wnt* gene loci displayed broadly conserved synteny across mammals, with limited variation observed among lineages.

### 3.5. Characterization of Tissue Expression Patterns of the Donkey Wnt Gene Family

**Figure 9:**
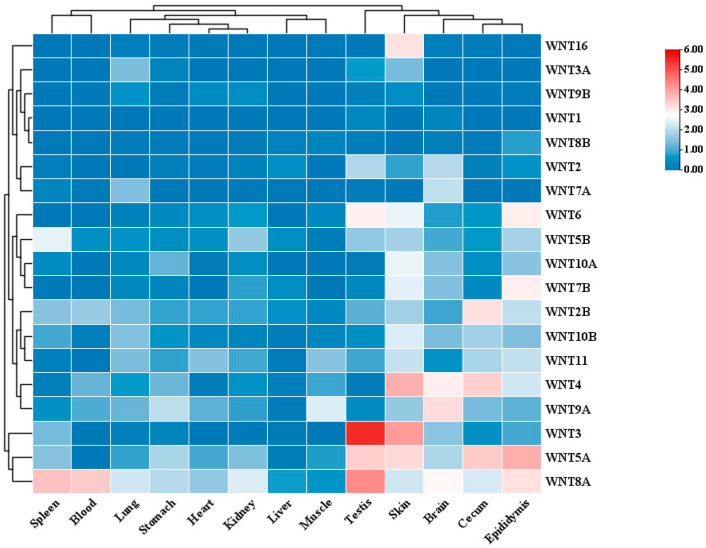
Expression of donkey *Wnt* family members in different tissues. Expression values were calculated from three independent biological replicates per tissue and are presented as log2(FPKM + 1). The color scale represents continuous log2(FPKM + 1) expression values, with gradients indicating low to high expression levels. Rows and columns were hierarchically clustered according to the similarity of *Wnt* expression profiles among genes and tissues, respectively.

Tissue expression profiling revealed distinct expression patterns among donkey *Wnt* genes across 13 adult tissues (Figure 9). Hierarchical clustering based on expression similarity grouped *Wnt* members and tissues according to their transcriptional profiles. *Wnt5a* and *Wnt8a* displayed relatively widespread expression patterns across multiple tissues, although their expression levels varied among tissues. In particular, *Wnt8a* showed the highest expression abundance in the testis, whereas *Wnt5a* exhibited relatively elevated expression in the epididymis. Several *Wnt* genes showed tissue-preferential expression patterns, including *Wnt3*, which was predominantly expressed in the testis, and *Wnt4*, which showed relatively higher expression in skin. Comparative analysis using horse and cattle expression datasets from the Bgee database further indicated that representative *Wnt* genes exhibited conserved tissue-associated expression patterns among mammals. For example, *Wnt3* showed detectable expression enrichment in reproductive-related tissues, while *Wnt4* and *Wnt5a* were also associated with epithelial and reproductive tissues in other mammalian species (Table S5). Collectively, these findings suggest that donkey *Wnt* family members exhibit tissue-preferred expression patterns, highlighting their potentially distinct roles in reproductive regulation, epithelial maintenance, and tissue homeostasis.

### 3.6. Integrated Functional Analysis and Prediction of Tissue-Associated Regulatory Networks of Donkey Wnt Genes

**Figure 10:**
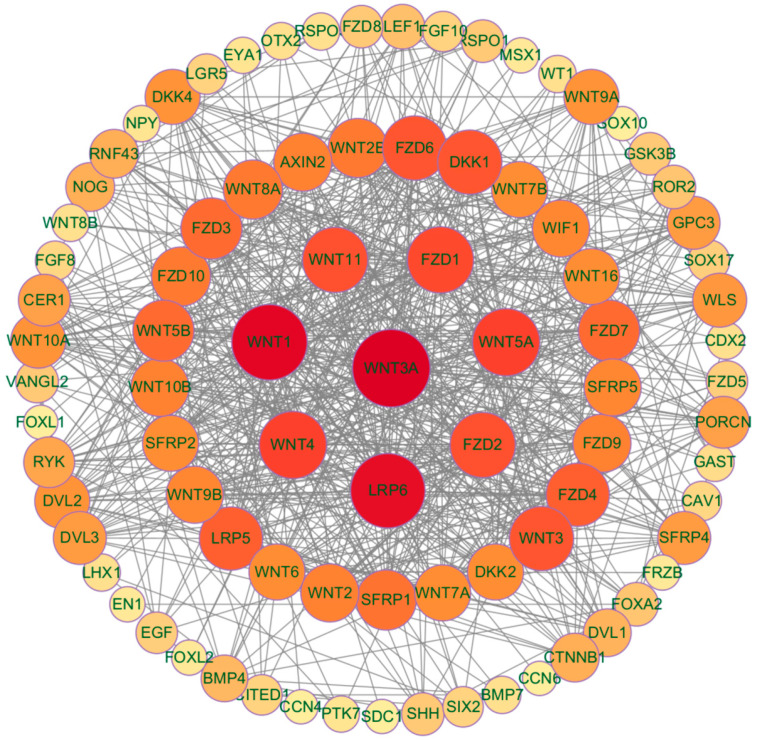
Protein–protein interaction (PPI) network of the donkey *Wnt* gene family and their associated signaling components. Node size and color gradient indicate connectivity, with larger and darker-colored nodes representing higher connectivity. Grey edges represent protein–protein interactions.

**Figure 11:**
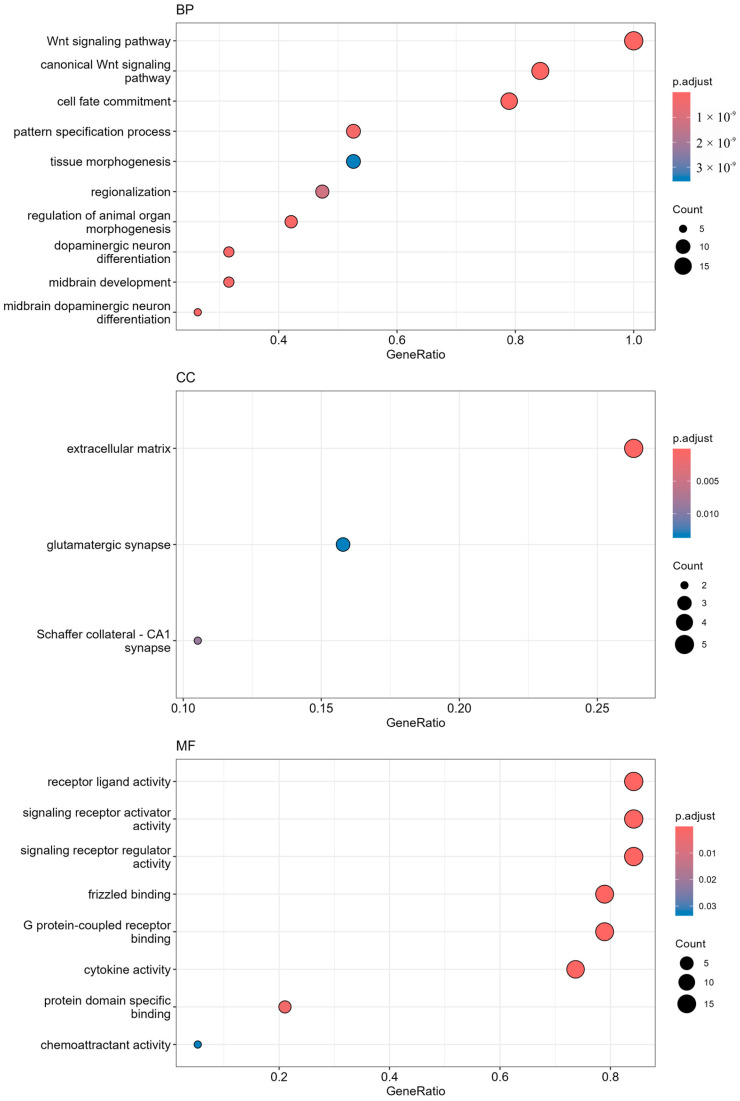
Gene Ontology (GO) enrichment analysis of donkey *Wnt* gene family members was performed using the DAVID online platform.

To investigate the potential functions of donkey Wnt proteins, GO enrichment analysis, predicted protein–protein interaction (PPI) analysis, subcellular localization prediction, and tissue expression profiling were integrated (Figure 10 and Figure 11, Table S6). The PPI network revealed potential associations between donkey Wnt proteins and conserved *Wnt* pathway components, including *FZD6*, *LRP5*, *DVL1/2*, *AXIN2*, *CTNNB1*, and extracellular regulators *DKK1* and *SFRP1* (Figure 10). GO enrichment analysis of the 19 donkey Wnt proteins indicated their involvement in developmental processes, cell fate determination, and canonical *Wnt* signaling pathways (Figure 11), with Cellular Component and Molecular Function terms mainly related to extracellular regions, receptor binding, and signaling activity, consistent with their roles as secreted signaling molecules.

Integration of tissue RNA-Seq profiles revealed coordinated expression patterns among several Wnt-associated interaction networks (Figure S5). *Wnt5a* showed relatively high expression in epididymis, accompanied by elevated expression of *FZD6*, *LRP5*, and *AXIN2*, suggesting a potential reproductive tissue-associated module. *Wnt4* exhibited preferential expression in skin, where *FZD1* and *DKK1* also showed higher expression levels. Core pathway components, including *DVL1/2*, *SFRP1*, and *CTNNB1*, displayed broader tissue expression patterns, consistent with their conserved roles in *Wnt* signaling regulation.

Cross-species comparison using horse and cattle expression resources further showed similar tissue expression tendencies for several genes, including *FZD6*, *SFRP1*, *LRP5*, and *CTNNB1* (Table S7). Subcellular localization prediction indicated that FZD and LRP proteins were mainly membrane-associated, *DKK1* and *SFRP1* were extracellular, *DVL1/2* and *AXIN2* were cytoplasmic, and *CTNNB1* showed both cytoplasmic and nuclear localization features (Table S6). Collectively, these analyses provide transcriptomic and functional annotation support for tissue-associated *Wnt* regulatory relationships in donkeys, although further experimental validation is required.

## 4. Discussion

### 4.1. Evolutionary Conservation and Molecular Characterization of the Wnt Gene Family in Equus asinus

A total of 19 *Wnt* genes were identified in this study, consistent with those reported in placental mammals such as human, mouse, and cattle, highlighting the evolutionary conservation of the *Wnt* signaling pathway [20,67,68]. The conserved gene repertoire and absence of lineage-specific expansion or loss highlight the stability of *Wnt* family organization in equids [3,69].

Protein physicochemical analysis revealed that *Equus asinus* Wnt proteins possess typical features of secreted signaling molecules, including high hydrophilicity and extracellular localization, consistent with their roles as intercellular signaling ligands [16,70]. Subfamily-specific differences in isoelectric point, protein length, and instability index indicate divergence in the physicochemical properties of Wnt proteins, which may influence their stability, molecular interactions, and regulatory functions [7,71]. Although Wnt proteins are structurally conserved, variations in surface charge distribution and post-translational modifications can influence interactions with Frizzled receptors and LRP5/6 co-receptors, thereby modulating canonical and non-canonical *Wnt* signaling pathways [6,72,73]. These physicochemical differences may contribute to variation in receptor binding, tissue-specific expression, and signaling regulation among *Wnt* family members [74].

In this study, SOPMA and AlphaFold were used to predict and compare the secondary structures of Wnt proteins. Wnt proteins were mainly composed of α-helices and random coils, with fewer β-sheets, which may be associated with conserved cysteine-rich regions and disulfide bond-mediated stabilization. Conserved cysteine residues form disulfide bonds that maintain Wnt folding and support Frizzled receptor binding and downstream signaling activation [7,8,64]. Amino acid composition analysis revealed residue preferences among secondary structural regions, with proline enrichment in regions with a lower α-helix content, suggesting its potential role in reducing helix stability and increasing local flexibility [64,65,66]. The negative correlation between α-helix and coil/loop regions reflects structural redistribution among different conformational states, while loop regions contribute to molecular recognition, conformational transitions, and protein interactions [75,76,77,78]. The positive association between α-helix content and AlphaFold confidence scores agrees with previous observations that ordered structures generally show higher prediction reliability, whereas flexible regions tend to have lower confidence values [46,47]. The weak correlation of β-sheets suggests that β-sheet variation may mainly represent local structural adjustments rather than changes in overall stability [79]. Although Wnt proteins share a conserved folding framework, subtle differences in secondary structure composition may affect flexible region distribution and receptor-binding properties. Differences between SOPMA- and AlphaFold-derived predictions likely result from methodological differences between sequence-based and structure-based approaches rather than true structural divergence [7,46].

Prediction of signal peptides and subcellular localization in *Equus asinus* Wnt proteins revealed that seven members contained classical signal peptides, whereas others, including Wnt4, Wnt5a, and Wnt16, showed weak or absent signal peptide predictions despite their predicted extracellular localization. This apparent discrepancy reflects the non-classical secretion mechanism of Wnt proteins, which relies not only on conventional signal peptides, but also on specialized trafficking machinery involving PORCN-mediated lipid modification and Wntless (WLS)-dependent transport [10]. Recent structural studies have demonstrated that WLS functions as a dynamic transporter rather than a passive carrier, and its conformational cycling facilitates the sequential capture, intracellular trafficking, and release of *Wnt* morphogens during secretion [80]. This WLS-mediated transport mechanism enables the extracellular delivery of Wnt proteins through a carrier-dependent pathway, providing a possible explanation for why some *Wnt* members lacking classical signal peptides can still exhibit extracellular localization [81]. Therefore, full-length protein-based localization predictions may better represent the functional extracellular distribution of *Wnt* ligands than signal peptide prediction alone [82].

Cross-species analysis of subcellular localization in human, cattle, horse, and donkey revealed a conserved extracellular localization pattern among all 19 *Wnt* orthologs. Extracellular localization showed the highest predicted scores, whereas other compartments exhibited low and relatively stable signals. This conserved pattern likely reflects functional constraints on Wnt proteins as secreted signaling molecules rather than sequence similarity alone [3,83,84].

### 4.2. Evolutionary Divergence, Structural Constraint and Genomic Stability of Wnt Genes

At the gene and protein levels, *Wnt* members exhibit strong evolutionary conservation, reflected by conserved exon organization and sequence architecture across metazoans [85]. Multiple sequence alignment demonstrated that this conservation is primarily maintained within the cysteine-rich core region (CRD), where conserved cysteine residues form a disulfide-bond network essential for maintaining *Wnt* structural integrity and mediating Frizzled receptor interactions [72]. In contrast, sequence variation was mainly concentrated in the N-terminal and C-terminal regions, which are generally more flexible and less constrained. These variable regions, together with their lower AlphaFold confidence scores, may represent structurally permissive segments that contribute to differences in protein conformation, ligand processing, and functional specialization among *Wnt* members [46,86].

Motif analysis further supports the conserved structural organization of Wnt proteins, with cysteine-rich motifs likely contributing to CRD stability through disulfide bond formation [72]. Although lacking cysteine residues, Motif9 was completely conserved across species, suggesting additional functional constraints and potential roles in conserved structural features or molecular interactions [2,85,86]. These findings support a modular *Wnt* architecture, in which conserved regions maintain signaling competence, while variable regions provide evolutionary flexibility for functional specialization.

At the evolutionary level, phylogenetic reconstruction provides insights into *Wnt* family diversification and conservation, with monophyletic clustering patterns suggesting that major *Wnt* diversification occurred during early vertebrate evolution, followed by functional conservation [3,47]. The heterogeneous branch length distributions among family members further indicate differential evolutionary constraints within the gene family [86]. Highly conserved members, such as *Wnt1* and *Wnt10a*, exhibited relatively short branches across vertebrate taxa, suggesting strong purifying selection. Although these genes showed relatively low expression levels in most adult donkey tissues, this does not necessarily indicate reduced biological importance, as *Wnt* genes often exhibit stage- and tissue-specific expression patterns, with essential functions during embryonic development and tissue specification [3,16]. Previous studies have demonstrated that *Wnt1* is involved in neural patterning and embryonic development, whereas *Wnt10a* contributes to ectodermal differentiation and tissue homeostasis, supporting the evolutionary constraint on these genes [87,88]. Although phylogenetic conservation cannot completely exclude the potential influence of neighboring genomic regions or linked regulatory elements, the conserved coding sequences and reported developmental functions of *Wnt1* and *Wnt10a* suggest that these genes themselves may also be subject to strong evolutionary constraints [89]. In contrast, *Wnt5a* and *Wnt7b* showed greater sequence divergence and longer branches, consistent with their roles in context-dependent non-canonical signaling pathways, where altered receptor interactions and functional specialization may contribute to lineage-specific diversification [90,91].

At the genomic level, synteny and comparative analyses further support the evolutionary constraints observed in *Wnt* genes. Despite their chromosomal dispersion, conserved syntenic relationships across mammals indicate long-term preservation of genomic organization under regulatory constraints. Notably, the conserved linkage of *Wnt3a*/*Wnt9a* and *Wnt3*/*Wnt9b* is consistent with the conserved genomic organization reported for *Wnt* family members in vertebrates. Such conserved gene arrangements may reflect the retention of ancestral genomic structures during vertebrate evolution [16,92]. The stable gene copy number and absence of recent lineage-specific expansions further suggest that dosage-sensitive regulation may have limited *Wnt* family restructuring, consistent with the need for the precise control of *Wnt* signaling during development [8,35,91].

### 4.3. Cross-Species Conservation and Regulatory Divergence of Wnt Gene Expression Patterns

Comparative expression analysis of representative *Wnt* genes in donkey, horse, and cattle (Table S5) revealed relatively conserved expression patterns among adult mammals, with some genes showing similar tissue distribution across species. However, differences in tissue enrichment levels were also observed among species. Cross-species comparisons further showed that representative *Wnt* genes are frequently expressed in reproductive- and epithelial-associated tissues [93,94,95], suggesting conserved functional constraints of *Wnt* genes in reproductive development, epithelial homeostasis, and establishment of the embryonic microenvironment [95,96]. Nevertheless, differences in predominant expression tissues among species indicate that evolutionary divergence of the *Wnt* gene family is more likely reflected at the level of transcriptional regulation rather than changes in protein-coding sequences [8,97].

These species-specific expression differences may partly arise from evolutionary changes in cis-regulatory regions [98,99]. *Wnt* transcription is controlled by complex regulatory networks involving promoters and distal enhancers, which integrate signals from *HOX* factors, *TCF/LEF* transcription factors, and hormone receptors to regulate spatiotemporal expression patterns [100,101,102]. Although *Wnt* coding sequences are highly conserved among mammals, non-coding regulatory regions show greater evolutionary flexibility, and enhancer gain, loss, or remodeling may contribute to interspecies differences in expression levels and tissue specificity [3,98,103,104]. In addition, epigenetic mechanisms may further shape tissue-specific *Wnt* expression, as enhancer-associated activation marks (e.g., H3K27ac enrichment) and chromatin accessibility are closely linked to *Wnt* transcriptional activity [105,106,107,108]. Notably, *Wnt8a* showed a conserved tendency toward neural and sensory tissue expression across species, suggesting a potentially conserved role in neurodevelopmental regulation, although further functional validation is required [107].

### 4.4. Tissue-Associated Expression Patterns and Potential Regulatory Modules of Donkey Wnt Genes

Based on tissue expression profiles, predicted interaction networks, and previous functional studies, several donkey *Wnt* members may be associated with biological processes underlying economically relevant traits, although further experimental validation is still required. Although PPI prediction cannot confirm direct protein–protein interactions, the consistency between predicted associations and coordinated tissue expression patterns provides support for the potential functions of specific *Wnt* regulatory modules.

Regarding reproductive-related traits, *Wnt5a* showed relatively high expression in the epididymis and showed similar tissue expression patterns with *FZD6*, *LRP5*, and *AXIN2*. Previous studies have demonstrated that *Wnt5a* participates in the regulation of Sertoli cell junctions, germ cell migration, and testicular development in mammals [109,110]. Through the non-canonical *Wnt* signaling pathway, *Wnt5a* contributes to the maintenance of testicular structural integrity and the regulation of reproductive processes [111,112]. In addition, *FZD6* and *LRP5*, as key receptor components of the *Wnt* signaling pathway, have been implicated in reproductive tissue development and cellular regulation, whereas *AXIN2* functions as a negative feedback regulator of Wnt/β-catenin signaling and reflects pathway activity [113]. Therefore, the coordinated expression of *Wnt5a*, *FZD6*, *LRP5*, and *AXIN2* in donkey reproductive tissues suggests that these genes may contribute to reproductive tissue maintenance through conserved *Wnt* signaling mechanisms.

Similarly, *Wnt3* exhibited relatively high expression in the testis and showed predicted associations with canonical Wnt/β-catenin signaling components, including *Wnt3a*, *LRP6*, and *CTNNB1*. Previous studies have shown that Wnt/β-catenin signaling is involved in mammalian gonadal development, germ cell formation and migration, cell proliferation, and establishment of the spermatogenic microenvironment [114,115,116,117]. Combined with the expression relationships observed between *Wnt3* and related signaling components in this study, these findings suggest that this signaling module may participate in testicular development and reproductive function regulation in donkeys [118,119].

For skin-related traits, *Wnt4* showed relatively high expression in skin tissue and showed similar tissue expression patterns with *FZD1* and *DKK1*. Previous studies have demonstrated that *Wnt4* contributes to epithelial differentiation, tissue patterning, epidermal renewal, and tissue regeneration [120,121,122], while *FZD1* and *DKK1* function as a *Wnt* receptor and a negative regulator of *Wnt* signaling, respectively, and are involved in skin appendage development, epidermal differentiation, and hair follicle formation [123,124]. Therefore, the *Wnt4*–*FZD1*–*DKK1* regulatory module may contribute to skin homeostasis and skin-related trait formation in donkeys.

However, differences in *Wnt* ligand expression patterns among species suggest that functional diversification of *Wnt* genes may primarily result from tissue-specific transcriptional regulation rather than alterations in the core signaling machinery [3]. Together with the spatial organization of extracellular ligands, membrane-associated receptors, and intracellular signaling mediators, these findings indicate that the donkey *Wnt* gene family maintains a conserved signaling framework while exhibiting tissue-specific regulatory characteristics. Collectively, *Wnt3*, *Wnt4*, and *Wnt5a* may serve as potential candidate genes for studies of reproductive and production-related traits in donkeys, providing valuable targets for future genetic and functional investigations; however, they should not yet be considered confirmed causal genes.

### 4.5. Study Limitations

We acknowledge several limitations of this genome-wide study. First, the donkey reference genome assembly used in this study (ASM1607732v2) [25] still contains limitations in some repetitive and complex genomic regions, which may have resulted in potentially missed or the inaccurate annotation of *Wnt* genes in poorly resolved regions. Second, the genome-wide identification and functional characterization analyses were mainly based on computational approaches, and predicted interaction networks were derived from computational databases and conserved functional associations; therefore, they should be interpreted as hypothesis-generating evidence rather than direct physical interactions. Third, the available RNA-Seq datasets were obtained from adult donkey tissues, representing a single developmental stage and limiting the assessment of developmental-stage-specific expression patterns. In addition, limited information regarding sex-specific expression differences restricted further evaluation of sex-dependent regulation of *Wnt* genes. Future studies incorporating improved genome assemblies, multi-stage transcriptomic datasets, and experimental validation will provide deeper insights into donkey *Wnt* gene evolution and biological functions.

## 5. Conclusions

This study systematically characterized the *Wnt* gene family in donkey, revealing strong conservation of gene structures, protein features, and evolutionary relationships across vertebrates. Although most *Wnt* members exhibited evolutionary conservation, *Wnt5a* and *Wnt7b* showed greater sequence divergence, suggesting lineage-specific functional specialization. In addition, *Wnt3*, *Wnt4*, and *Wnt5a* displayed tissue-preferential expression patterns associated with reproductive and skin-related processes, highlighting their potential roles in donkey biological traits. These findings suggest that *Wnt3*, *Wnt4*, and *Wnt5a* may represent potential candidate genes for the further investigation of donkey development and economically important traits.

## Figures and Tables

**Table 1:** Basic genomic information of *Wnt* gene family members in donkey.

Gene Name	Protein Accession	Chromosome (Chr)	Gene Start (bp)	Gene End (bp)	Number of Exons	Chain
***Wnt1***	XP_014705617	22	19,258,419	19,261,521	6	−
***Wnt2***	XP_014693894	1	27,722,861	27,765,466	5	+
***Wnt2b***	XP_014692714	16	6,827,451	6,839,753	5	−
***Wnt3***	XP_044601377	13	46,626,362	46,673,292	4	+
***Wnt3a***	XP_044634035	9	64,366,391	64,408,329	6	−
***Wnt4***	XP_044625891	5	17,829,876	17,856,193	6	−
***Wnt5a***	XP_014724228	21	64,215,110	64,234,004	5	−
***Wnt5b***	XP_044611473	22	29,317,000	29,333,614	7	−
***Wnt6***	XP_014708053	19	19,956,666	19,969,618	4	−
***Wnt7a***	XP_014693941	21	94,235,519	94,299,284	4	+
***Wnt7b***	XP_014719938	2	4,362,347	4,411,634	4	+
***Wnt8a***	XP_014709282	12	15,945,112	15,950,939	6	+
***Wnt8b***	XP_070341426	2	75,539,944	75,564,118	6	−
***Wnt9a***	XP_070374131	9	64,434,807	64,480,984	4	+
***Wnt9b***	XP_070339511	13	46,582,249	46,602,099	4	−
***Wnt10a***	XP_044607450	19	19,938,119	19,950,245	4	−
***Wnt10b***	XP_014705507	22	19,268,732	19,274,529	5	+
***Wnt11***	XP_014711321	20	69,033,532	69,054,470	7	+
***Wnt16***	XP_014694588	1	24,463,002	24,474,193	4	−

**Table 2:** Physicochemical properties of *Wnt* gene family proteins.

Gene Name	Number of Amino Acids	Molecular Weight (kD)	Theoretical pI	Instability Index	Aliphatic Index	Grand Average of Hydropathicity
***Wnt1***	363	40.15	9.41	62.18	72.04	−0.369
***Wnt2***	360	40.44	9.24	42.81	62.89	−0.422
***Wnt2b***	393	44.06	9.37	50.45	67.81	−0.448
***Wnt3***	355	39.67	7.46	44.66	69.24	−0.359
***Wnt3a***	478	52.37	8.25	56.67	59.81	−0.399
***Wnt4***	449	48.10	9.56	61.35	59.82	−0.587
***Wnt5a***	380	42.34	8.83	38.93	67.26	−0.303
***Wnt5b***	557	62.56	9.37	52.60	64.65	−0.464
***Wnt6***	392	42.74	9.24	43.42	65.05	−0.443
***Wnt7a***	349	38.97	9.05	35.80	67.94	−0.393
***Wnt7b***	370	41.37	9.39	50.84	60.97	−0.631
***Wnt8a***	370	41.39	8.30	57.10	65.70	−0.378
***Wnt8b***	351	38.74	8.89	53.67	65.38	−0.387
***Wnt9a***	365	40.44	9.12	51.21	69.04	−0.395
***Wnt9b***	358	39.06	9.15	54.46	72.35	−0.253
***Wnt10a***	417	46.22	9.42	62.81	70.00	−0.433
***Wnt10b***	389	43.00	9.32	51.74	71.98	−0.409
***Wnt11***	540	57.96	9.34	51.34	65.37	−0.495
***Wnt16***	405	44.72	9.16	42.94	71.56	−0.372

**Table 3:** Secondary structure prediction and subcellular localization of donkey *Wnt* gene family proteins.

Gene Name	Proportion of Secondary Structure Distribution				Subcellular Localization
	Alpha Helix	Extended Strand	Beta Turn	Random Coil	
*Wnt1*	37.47	12.12	1.93	48.48	Extracellular
*Wnt2*	39.72	9.44	3.06	47.78	Extracellular
*Wnt2b*	38.17	8.91	2.04	50.82	Extracellular
*Wnt3*	38.87	11.27	2.54	47.32	Extracellular
*Wnt3a*	33.26	10.88	2.30	53.56	Extracellular
*Wnt4*	25.84	8.69	3.79	61.68	Extracellular
*Wnt5a*	38.42	10.79	1.84	48.95	Extracellular
*Wnt5b*	31.24	8.98	4.67	55.11	Extracellular
*Wnt6*	37.50	9.95	2.30	50.25	Extracellular
*Wnt7a*	39.26	10.32	2.01	48.41	Extracellular
*Wnt7b*	34.05	9.46	2.97	53.52	Extracellular
*Wnt8a*	33.51	12.16	2.70	51.63	Extracellular
*Wnt8b*	32.19	11.40	2.28	54.13	Extracellular
*Wnt9a*	40.00	9.32	1.92	48.76	Extracellular
*Wnt9b*	38.27	10.89	2.79	48.05	Extracellular
*Wnt10a*	32.37	8.63	1.68	57.32	Extracellular
*Wnt10b*	37.28	9.00	1.80	51.92	Extracellular
*Wnt11*	26.67	8.15	1.67	63.51	Extracellular
*Wnt16*	36.79	10.12	1.98	51.11	Extracellular

## Data Availability

The data used in this study were downloaded from the ENSEMBL database (*Equus_asinus*—Ensembl genome browser 115) on 10 June 2025.

## Supplementary Materials

The following supporting information can be downloaded at: https://www.mdpi.com/article/10.3390/biology15151323/s1, Supplementary Figures: Supplementary analyses of Wnt protein characteristics, phylogenetic relationships, and expression profiles; Supplementary Tables: Supplementary analyses of structural characteristics, localization features, conserved motifs, and expression profiles of Wnt proteins and Wnt-related genes. Supplementary Material File: Multiple Sequence Alignment of the Donkey *Wnt* Gene Family.pdf.

## Abbreviations

The following abbreviations are used in this manuscript:

Wnt	Wingless-Related Integration Site
CRD	Cysteine-Rich Domain
CDS	Coding Sequence
UTR	Untranslated Region
HMMER	Hidden Markov Model-Based Sequence Analysis Tool
Pfam	Protein Families Database
CDD	Conserved Domain Database
SMART	Simple Modular Architecture Research Tool
MEME	Multiple Em for Motif Elicitation
ML	Maximum Likelihood
BI	Bayesian Inference
MDS	Multidimensional Scaling
PPI	Protein–Protein Interaction
GO	Gene Ontology
RNA-Seq	RNA Sequencing
SRA	Sequence Read Archive
SP_score	Signal Peptide Probability Score
AlphaFold	Artificial Intelligence-Based Protein Structure Prediction System
pLDDT	Predicted Local Distance Difference Test
FZD	Frizzled Receptor
LRP	Low-Density Lipoprotein Receptor-Related Protein
WLS	Wntless
PORCN	Porcupine O-Acyltransferase
AXIN	Axis Inhibition Protein
CTNNB1	Catenin Beta 1
DKK	Dickkopf-Related Protein
SFRP	Secreted Frizzled-Related Protein
