# Supplementary material for: Genome-Wide Identification and Characterization of the Wnt Gene Family in Donkey (Equus asinus): Phylogenetic Analysis and Expression Profiling
Source: Biology (Basel). 2026 Aug 6;15(15):1323. doi: 10.3390/biology15151323 (PMC13465090; doi:10.3390/biology15151323)
Supplement: Supplementary file 1 [file biology-15-01323-s001.zip › Supplementary Figures.pdf]

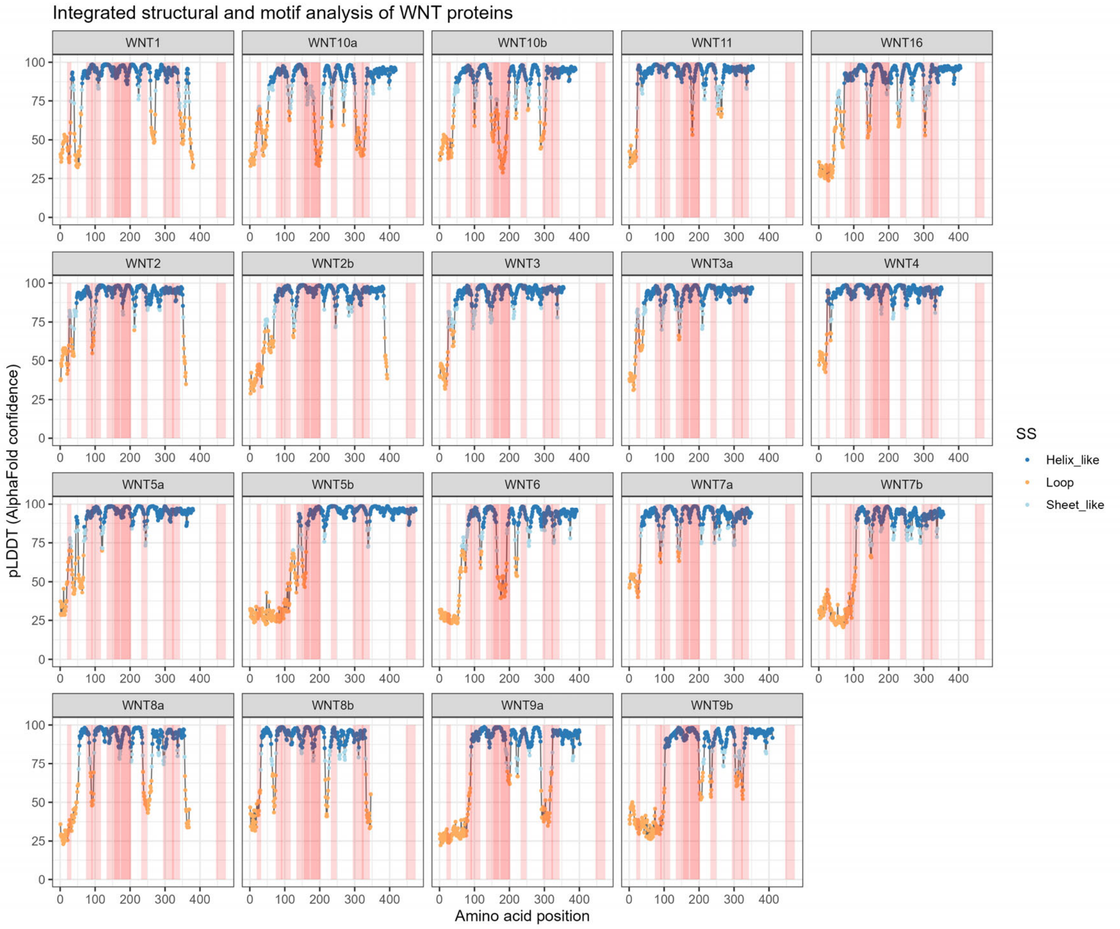

Supplementary Figure S1. Distribution of pLDDT confidence and secondary structural motifs along WNT protein amino acid sequences

The x-axis represents amino acid positions across the full-length WNT polypeptide, while the y-axis indicates AlphaFold pLDDT structural confidence scores. Three types of secondary structures are distinguished by colored dots: helix-like regions (dark blue), sheet-like regions (light blue), and flexible loop regions (orange). Shaded red vertical bands denote evolutionarily conserved ligand-binding motifs of WNT proteins, which mediate binding to the cysteine-rich domain (CRD) of Frizzled receptors.

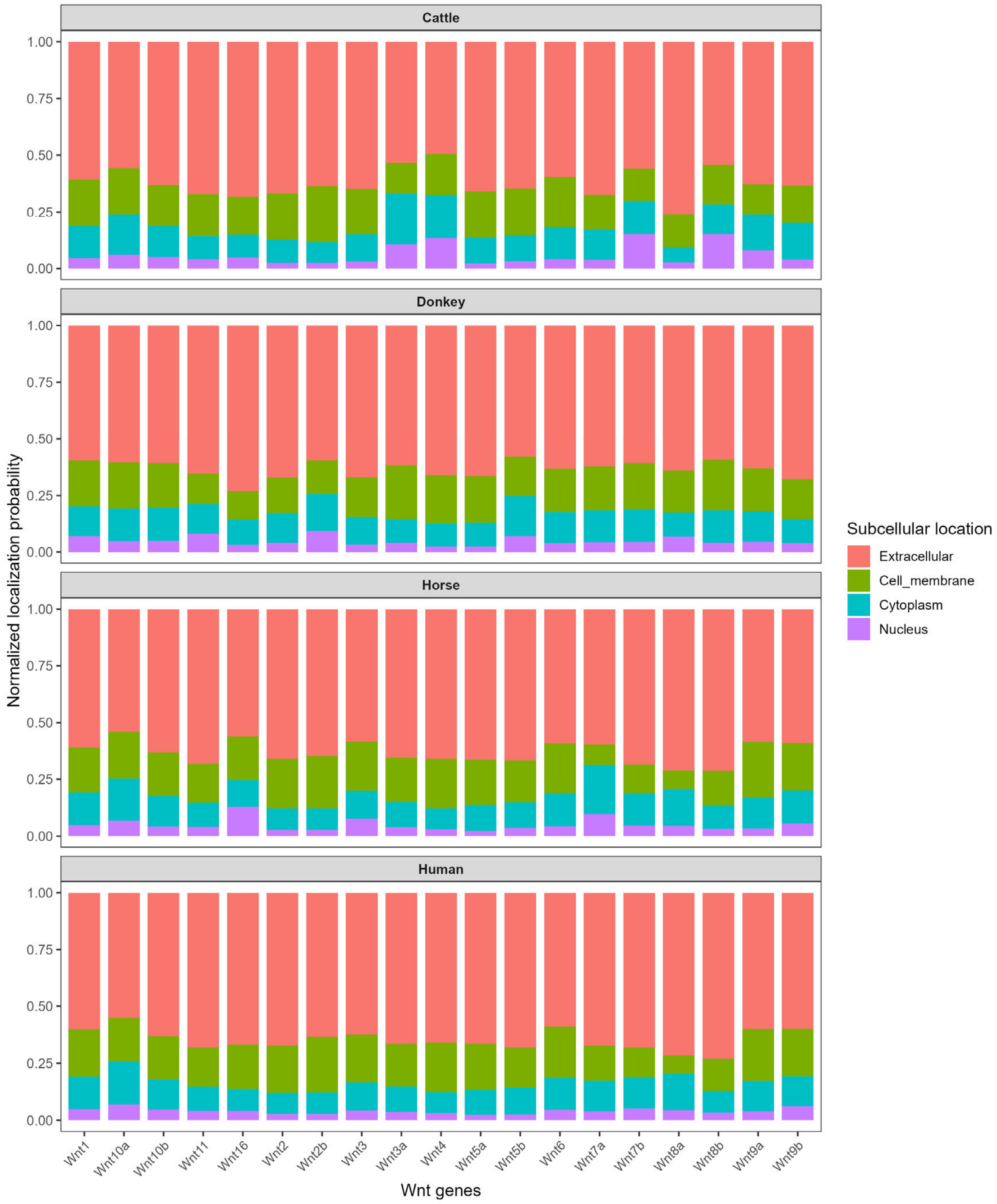

Supplementary Figure S2. Predicted subcellular localization probability of WNT proteins across four species

Each stacked bar corresponds to one WNT gene, and the vertical axis represents normalized localization probability (0–1). Four subcellular compartments are distinguished by different colors: Extracellular (red), Cell membrane (green), Cytoplasm (cyan), Nucleus (purple).

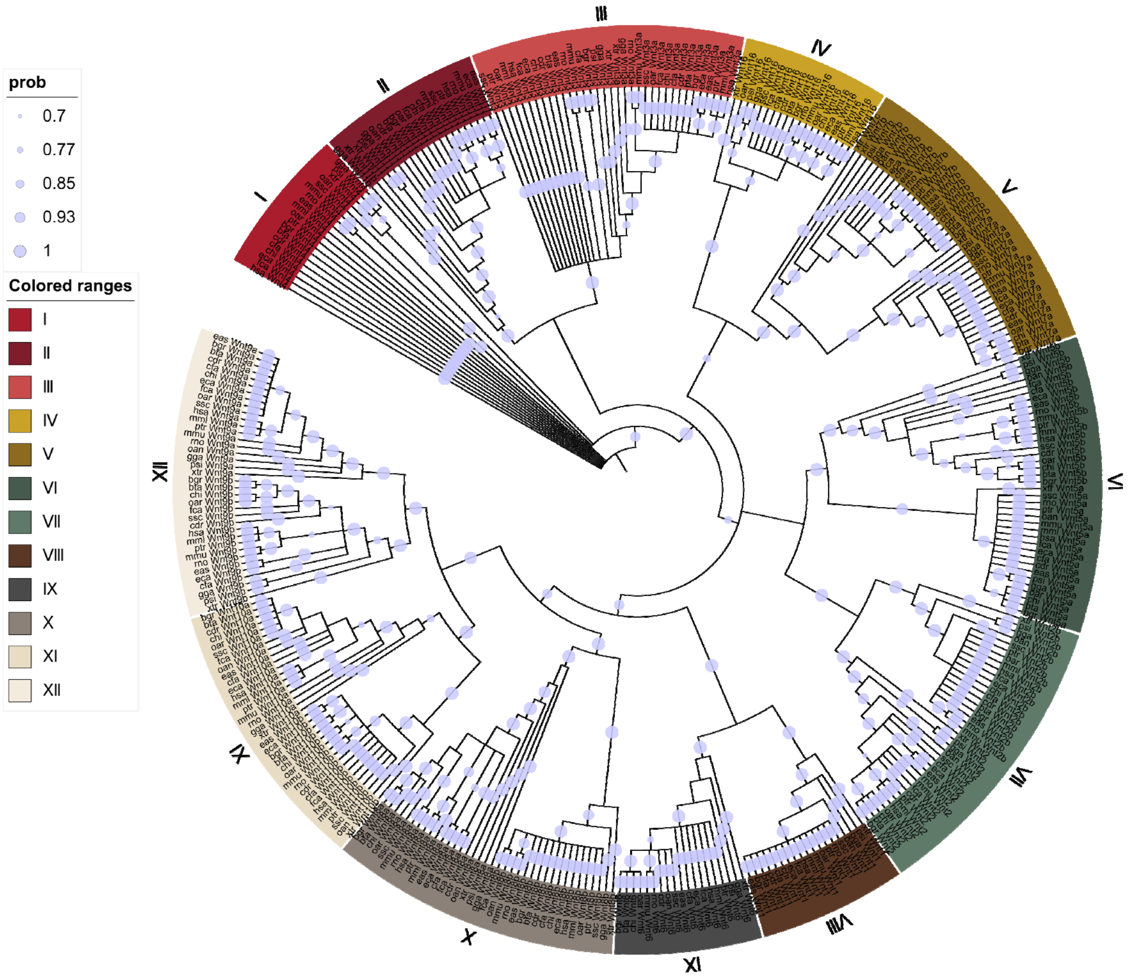

Supplementary Figure S3. Bayesian inference phylogenetic tree of WNT family proteins

The phylogenetic tree was constructed based on full-length amino acid sequences of WNT proteins. Posterior probability values are labeled at each branch node to evaluate tree reliability. Different colored branches represent distinct WNT subfamilies, and species labels are marked at the branch terminals.

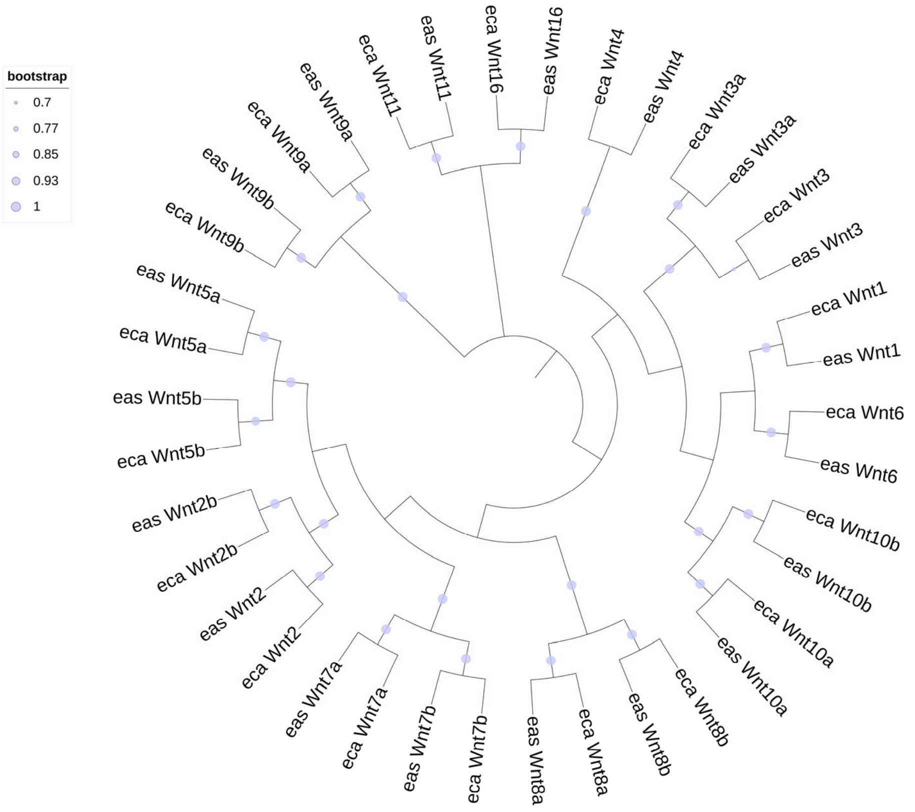

Supplementary Figure S4. Maximum-likelihood phylogenetic tree of donkey and horse Wnt genes.

The phylogenetic tree was inferred using full-length amino acid sequences of Wnt proteins from donkey and horse based on the Maximum Likelihood method in MEGA12. Bootstrap values were estimated from 1000 replicates and are shown at nodes with support values ≥70%. The tree illustrates the evolutionary relationships and conservation of Wnt family members between the two equid species.

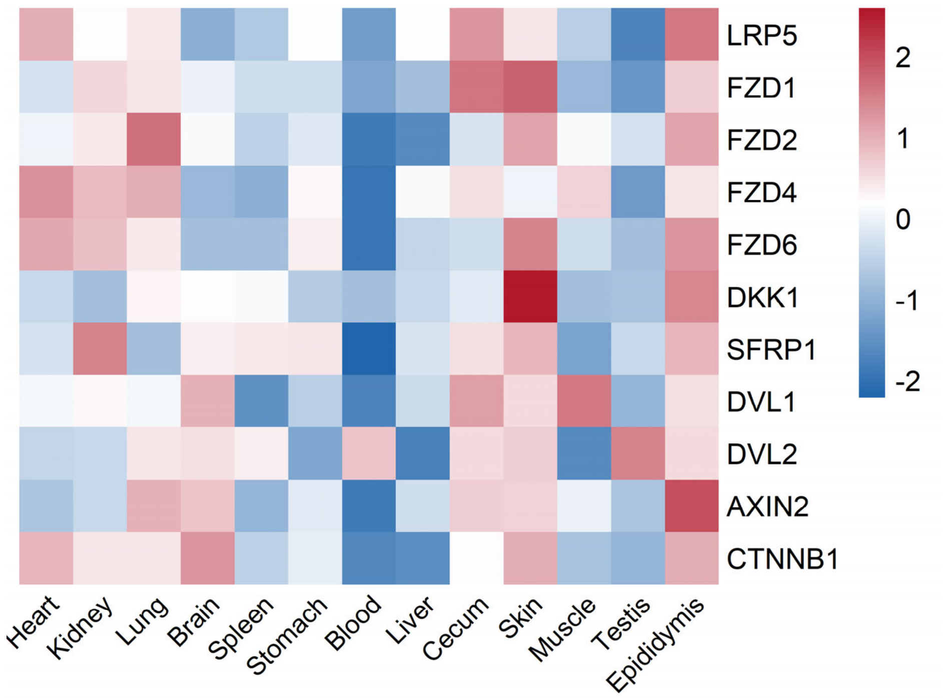

Supplementary Figure S5. Expression heatmap of Wnt signaling interacting proteins in different donkey tissues.

Rows represent interacting proteins of the Wnt pathway, and columns indicate various tissue samples. The colour gradient represents normalized gene expression values; red denotes higher expression, and blue denotes lower expression.
