# Supplementary material for: Genome-Wide Identification and Characterization of the Wnt Gene Family in Donkey (Equus asinus): Phylogenetic Analysis and Expression Profiling
Source: Biology (Basel). 2026 Aug 6;15(15):1323. doi: 10.3390/biology15151323 (PMC13465090; doi:10.3390/biology15151323)
Supplement: Supplementary file 1 [file biology-15-01323-s001.zip › Supplementary File. Multiple Sequence Alignment of the Donkey Wnt Gene Family.pdf]

```
            1
eas_Wnt4    M............................................................................................
eas_Wnt5a   MKK.....................................................SIGIL................................
eas_Wnt5b   MTKGKEQVSSLICPLLAPHTCLLVPLPQLRPQEEKPKGGGEQAVAQLPWILLRRPSSLGGLRTKISPRLMTQITCRPLPESLETQKRGSSAGA
eas_Wnt2    .............................................................................................
eas_Wnt2b   MLR..................................PSGAEE..................AAQLPPRRV..............RAPVPAPAP
eas_Wnt3    M............................................................................................
eas_Wnt3a   M............................................................................................
eas_Wnt7a   M............................................................................................
eas_Wnt7b   M............................................................................................
eas_Wnt16   MLS..................................AGGKKARDNL............VLGTDLWPGGL.........LGGESRRGVTGAGG
eas_Wnt11   M............................................................................................
eas_Wnt6    MFS..................................GTHQHH.................LYSACLPRAV.........QAQE..........
eas_Wnt10a  MGS..................................THPCPR.................LRLRPRP..........................
eas_Wnt10b  MLE............................................................EPRP..........................
eas_Wnt1    MG...........................................................................................
eas_Wnt8a   M...........................................................LPSVPLPGKS.......................
eas_Wnt8b   M..............................................................................EHLQ..........
eas_Wnt9a   MGC..................................DL......................................................
eas_Wnt9b   M............................................................................................

                                             10                                                      20
eas_Wnt4    .............SP......RSCLRS......LRLLVFAVFS.............................................AAASN
eas_Wnt5a   .............SPGVALGTAGSAMSS....KFFLMALAIFFSFAQ.......................................VVIEANS
eas_Wnt5b   LFWKPFIPGV.FRSPGPWGGLRPTMPS......LLLLAAALLSSWAQ.......................................LPAEASS
eas_Wnt2    ...........MNAP......LGGIWLW.....LPLLLTWLTP.............................................EVSSS
eas_Wnt2b   RPAAP..DGS.PASP......RLSLACL.....LLLLLLTLPA.............................................RVDTS
eas_Wnt3    .............EP.................HLLGLLLGLLLCGTR.......................................VLAGYPI
eas_Wnt3a   .............AP.....................LGYFLFLCGLK......................................QALGSYPI
eas_Wnt7a   ...........NRKA......RRCLGH......LFL....................................................SLGMV
eas_Wnt7b   ...........AQPPSLDH..AQKLSQ......VDFLRVSLLW............................................RPVREA
eas_Wnt16   LHA.....GAMDRAALLGLS.RLCA........LWAALLTLFPC............................................GAQGN
eas_Wnt11   ...........RARP......QLCEAL......LFALALQTGV.............................................CYGIK
eas_Wnt6    ...........KDTAVKGKTKNTCASH.............................................................SLDRL
eas_Wnt10a  ...........QPRP......ALCALL......FFLLLLAAAV.............................................PRSAP
eas_Wnt10b  ...........RPPP......SGLVGL......LFLALC.................................................SRALS
eas_Wnt1    .HW........ALLP......GWVSAT......LLLALAALPAA.........................................LAANSSGR
eas_Wnt8a   ...........FLPPL.....TLCQGSSRYLIPIHHCLTFSLFG.................................................
eas_Wnt8b   ...........KNAKA.....RQTQGD..................................................................
eas_Wnt9a   ......LKGVLGEGPV.....AIGVPE.......TVLTLQLVGEQVQGGSLVCKGP..........RPGWFFCSPH..............GGL
eas_Wnt9b   ...........RPPP......ALALAA......LCLLALPAAAAAAYFGSVPAAPPAEPPQPPLRRPP.....SPHLPHVHRDCHPSSSLSQP

                                            30             40        50        60        70        80
eas_Wnt4    WLYLA.......................KLSSVGSIS.....EEETCEKLKGLIQRQVQMCKRNLEVMDSVRRGAQLAIEECQYQFRNRRWNC
eas_Wnt5a   WWSLGMNNPV..................QMSEVYIIG.....AQPLCSQLAGLSQGQKKLCHLYQDHMQYIGEGAKTGIKECQYQFRHRRWNC
eas_Wnt5b   WWSLAM.NPV..................QRPEMFIIG.....AQPVCSQLPGLSPGQRKLCQLYQEHMAYIGEGAKTGIKECQYQFRQRRWNC
eas_Wnt2    WWYMR.............................ATG...GASRVMCDNVPGLVSRQRQLCHRHPDVMRAIGLGVAEWTAECQHQFRQHRWNC
eas_Wnt2b   WWYIG.............................ALG.....ARVICDNIPGLVSRQRQLCQRYPDIMRSVGEGAREWIRECQHQFRHHRWNC
eas_Wnt3    WWSLALG.......................QQYTSLG....SQPLLCGSIPGLVPKQLRFCRNYIEIMPSVAEGVKLGIQECQHQFRGRRWNC
eas_Wnt3a   WWSLAVG.......................PQYSSLG....TQPILCASIPGLVPKQLRFCRNYVEIMPSVAEGVKISIQECQHQFRGRRWNC
eas_Wnt7a   YLRIG.......................GFSSVVALG.....ASIICNKIPGLAPRQRAICQSRPDAIIVIGEGSQMGLDECQFQFRNGRWNC
eas_Wnt7b   W.........................................SAVVCRG.PGRQHHLQQDSWLGPAAARHLPEPAR.....CHH....RDWGG
eas_Wnt16   WMWLGI.........................ASFGVP.....EKLGCANLP.LNSRQKELCKRKPYLLPSIREGARLGIQECRNQFRHERWNC
eas_Wnt11   WLALS.......................KTPAALALN.....QTQHCKQLEGLVSAQVQLCRSNLELMRTVVHAAREATKACRRAFSDMRWNC
eas_Wnt6    FSYVTREGG..................RAVGSPLVMD.....PTSICRKARRLAGRQAELCQAEPEVVAELARGARLGVRECQFQFRFRRWNC
eas_Wnt10a  NDILGL......................RLPPEPVLN.....ANTVCLTLPGLSRRQMEVCVRHPDVAASAIQGIQIAIHECQHQFRDQRWNC
eas_Wnt10b  NEILGL......................KLPGEPPLT.....ANTVCLTLSGLSKRQLGLCLRSPDVTASALQGLHIAVHECQHQLRDQRWNC
eas_Wnt1    WWGIVN......................VASSTNLLTDSKSLQLVLEPSLQLLSRKQRRLIRQNPGILHSVSGGLQSAVRECKWQFRNRRWNC
eas_Wnt8a   ...........................RSVNNFLITGP.......................KAYLTYTTSVALGAQSGIEECKFQFAWERWNC
eas_Wnt8b   ..........................RRSVNNFLMTGP.......................KAYLIYSSSVAAGAQSGIEECKYQFAWDRWNC
eas_Wnt9a   WG..GAHPGLSVPRLTGSEPLTILP..LTLEPEAAAH....AHYKACDRLK.LERKQRRMCRRDPGVAETLVEAVSMSALECQYQFRFERWNC
eas_Wnt9b   LKVTGL.PPLALSSLTGREVLTPFPGLGTAAAPAQGG....AHLKQCDLLK.LSRRQKQLCRREPGLAETLQEAAHLSLLECQFQFRHERWNC

            90                         100       110       120       130       140
eas_Wnt4    STLD..................SLPVFGKVVTQGTREAAFVYAISSAGVAFAVTRACSSGELEKCGCDRTVHGV...................
eas_Wnt5a   STVD..................NTSVFGRVMQIGSRETAFTYAVSAAGVVNAMSRACREGELSTCGCSRAARPK...................
eas_Wnt5b   STVD..................NTSVFGRVMQIGSRETAFTYAVSAAGVVNAISRACREGELSTCGCSRTARPK...................
eas_Wnt2    NTLD.................RDHSLFGRVLLRSSRESAFVYAISSAGVVFAITRACSQGELRSCSCDPKKKGTAK.................
eas_Wnt2b   TTLD.................RDHTVFGRVMLRSSREAAFVYAISSAGVVHAITRACSQGELSVCSCDPYTRGRHH.................
eas_Wnt3    TTID.................DSLAIFGPVLDKATRESAFVHAIASAGVAFAVTRSCAEGTSTICGCDSHHKGP...................
eas_Wnt3a   TTVN.................NSLAIFGPVLDKATRESAFVHAIASAGVAFAVTRSCAEGSAAICGCSGRQQGS...................
eas_Wnt7a   SALG..................ERTVFGKELKVGSREAAFTYAIIAAGVAHAITAACTQGNLSDCGCDKEKQGQYH.................
eas_Wnt7b   GADGHQRVPVPVPLWTLELLRPRREDRLRARAPRSREAAFTYAITAAGVAHAVTAACSQGNLSNCGCDREKQGYYN.................
eas_Wnt16   RVAAT..AP...........PGTSPIFGYELTSGTKETAFIYAVMAAGLVHSVTRSCSAGNMTECSCDTTLQNGGS.................
eas_Wnt11   SSIE..................LAPNYLLDLERGTRESAFVYALSAAAISHAIARACTTGDLPGCSCGPVPGEP...................
eas_Wnt6    SSHS....................KAFGRILQQDIRETAFVFAITAAGASHAVTQACSMGELLQCGCQAPRGRAPP...............RP
eas_Wnt10a  SSLET...............RNKIPYESPIFSRGFRESAFAYAIAAAGVVHAVSNACALGKLKACGCDASRRGDEEAFRRKLHRLQLDALQRG
eas_Wnt10b  SALEG...............GGRLPHHSAILKRGFRESAFSFSMLAAGVMHAVATACSLGKLVSCGCDWKGSGEQDRLRAKL..LQLQALSRG
eas_Wnt1    PTAS..................GPHLFGKIVNRGCRETAFIFAITSAGVTHSVARSCSEGSIESCTCDYRRRGP...................
eas_Wnt8a   PENA..................LQLSTHNRLRSATRETSFIHAISSAGVMYTITKNCSMGDFENCGCDESKNGKT..................
eas_Wnt8b   PERA..................LQLSSHGGLRSANRETAFVHAISSAGVMYTLTRNCSLGDFDNCGCDDSRNGQL..................
eas_Wnt9a   TLEG....................RYRASLLKRGFKETAFLYAISSAGLTHALAKACSAGRMERCTCDEAPDLE...................
eas_Wnt9b   SLEG......................RTALLKRGFKETAFLYAVSSAALTHTLARACSAGRMERCTCDDSPGLE...................
```

```
                                        150       160       170       180       190       200       210
eas_Wnt4      ...................SPQGFQWSGCSDNIAYGVAFSQSFVDVRERSK.....GASSSRAL..MNLHNNEAGRKAILTHMRVECKCHGVS
eas_Wnt5a     ..................DLPRDWLWGGCGDNIDYGYRFAKEFVDARERERIHAKGSYESARIL..MNLHNNEAGRRTVYNLADVACKCHGVS
eas_Wnt5b     ..................DLPRDWLWGGCGDNVEYGYRFAKEFVDAREREKNFAKGSEEQGRVL..MNLQNNEAGRRAVYKMADIACKCHGVS
eas_Wnt2      ..................DSKGTFDWGGCSDNIDYGIKFARAFVDAKERKG.......KDARAL..MNLHNNRAGRKAVKRFLKQECKCHGVS
eas_Wnt2b     ..................DQRGDFDWGGCSDNIHYGVRFAKAFVDAKEKRL.......KDARAL..MNLHNNRCGRTAVRRFLKLECKCHGVS
eas_Wnt3      ...................PGEGWKWGGCSEDADFGVLVSREFADARENR........PDARSA..MNKHNNEAGRTTILDHMHLKCKCHGLS
eas_Wnt3a     ...................PGEGWKWGGCSEDIEFGGMVSREFADARENR........PDARSA..MNRHNNEAGRQAIASHMHLKCKCHGLS
eas_Wnt7a     ...................RDEGWKWGGCSADIRYGIGFAKVFVDAREIK........QNARTL..MNLHNNEAGRKILEENMKLECKCHGVS
eas_Wnt7b     ...................QAEGWKWGGCSADVRYGIDFSRRFVDAREIK........KNARRL..MNLHNNEAGRKVLEERMKLECKCHGVS
eas_Wnt16     ...................ASEGWHWGGCSDDVQYGMWFSRKFLDVPNKNT.....TGKESKILLAMNLHNNEAGRQAVAKLMSVDCRCHGVS
eas_Wnt11     ...................PGPGNRWGGCADNLSYGLLMGAKFSDAPMKVKK....TGSQANKL..MRLHNSEVGRQALRASLEMKCKCHGVS
eas_Wnt6      PGLP.GTPGP.PGPAGSPDGSAAWEWGGCGDDVDFGDEKSRLFMDARHKRG......RGDIRAL..VQLHNNEAGRLAVRSHTRTECKCHGLS
eas_Wnt10a    KGLSHGVPEHPALPPASPGLQDSWEWGGCSPDVGFGERFSKDFLDSREPH........RDIHAR..MRLHNNRVGRQAVMENMRRKCKCHGTS
eas_Wnt10b    KSFPHSLPSPGPGSGPSPGPQDTWEWGGCNHDMDFGEKFSRDFLDSREAP........RDIQAR..MRIHNNRVGRQVVTENLKRKCKCHGTS
eas_Wnt1      ...................GGPDWHWGGCSDNIDFGRLFGREFVDSGEKG........RDLRFL..MNLHNNEAGRTTVFSEMRQECKCHGMS
eas_Wnt8a     ...................GGHGWIWGGCSDNVEFGERISKLFVDSLEKG........KDARAL..MNLHNNRAGRLAVRATMKRTCKCHGIS
eas_Wnt8b     ...................GGQGWLWGGCSDNVGFGEAISKQFVDALETG........QDARAA..MNLHNNEAGRKAVKGTMKRTCKCHGVS
eas_Wnt9a     ...................NREAWQWGGCGDNLKYSSKFVKEFLG.RRSS........KDLRAR..VDFHNNLVGVKVIKAGVETTCKCHGVS
eas_Wnt9b     ...................SRQAWQWGVCGDNLKYSTKFLSNFLGPKRGS........KDLRAR..ADAHNTHVGIKAVKSGLRTTCKCHGVS

                    220       230       240       250                 260       270       280       290
eas_Wnt4      GSCEVKTCWRAVPPFRQVGHALKEKFDGATEVEPRRVGSSR...A....LVPR....NAQFKP...HTDEDLVYLEPSPDFCEQDMRSGVLGT
eas_Wnt5a     GSCSLKTCWLQLADFRKVGDALKEKYDSAAAMRLNSRGK..........LVQV....NSRFNS...PTTQDLVYIDPSPDYCVRNESTGSLGT
eas_Wnt5b     GSCSLKTCWLQLAEFRKVGDQLKEKYDSAAAMRITRKGK..........LELV....NSRFNQ...PTPEDLVYVDPSPDYCLRNETTGSLGT
eas_Wnt2      GSCTLRTCWLAMADFRKTGDYLWRKYNGAIQVVMNQDGTG.........FTVA....NKKFKK...PTKNDLVYFENSPDYCIRDRDAGSLGT
eas_Wnt2b     GSCTLRTCWRALSDFRRTGDYLRRRYDGAVQVTATQDGAN.........FTAA....RQGYRR...ATRTDLVYFDNSPDYCVLDKAAGSLGT
eas_Wnt3      GSCEVKTCWWAQPDFRAIGDFLKDKYDSASEMVVEKHRESR...GWVETLRAK....YALFKP...PTERDLVYYENSPNFCEPNPETGSFGT
eas_Wnt3a     GSCEVKTCWWSQPDFRAIGDFLKDKYDSASEMVVEKHRESR...GWVETLRPR....YTHFKV...PTERDLVYYEASPNFCEPNPETGSFGT
eas_Wnt7a     GSCTTKTCWTTLPQFRELGYVLKDKYNEAVHVEPVRASRNK...RPTFLKIKK....PLSYRK...PMDTDLVYIEKSPNYCEEDPVTGSVGT
eas_Wnt7b     GSCTTKTCWTTLPKFREVGHLLKEKYNAAVQVEVVRASRLR...QPTFLRIKQ....LRSYQK...PMETDLVYIEKSPNYCEEDAATGSVGT
eas_Wnt16     GSCAVKTCWKTMSSFEKIGHLLKDKYENSVQISDKIKRKMR........RREK....DQRKIP...IHKEDLLYINKSPNYCVEDKKLGIPGT
eas_Wnt11     GSCSIRTCWKGLQELRDVAADLKTRYLSATKVVHRPMGTRK...H....LVPK....DLDIRP...VKDSELVYLQSSPDFCMKNEKVGSHGT
eas_Wnt6      GSCALRTCWQKLPPFREVGARLLERFHGASRVMGTNDGKA.........LLPA....VRTLKP...PGRADLLYAADSPDFCAPNRRTGSPGT
eas_Wnt10a    GSCQLKTCWQVTPEFRAVGALLRSRFHRATLIRPHNRNGGQLEPGPAGAPSPA.PGVPGPRRR...ASPADLVYFEKSPDFCEREPRLDSAGT
eas_Wnt10b    GSCQFKTCWRAAPEFRAVGAALRERLGRAIFIDTHNRNSGAFQ..............PRLRPR...RLSGELVYFEKSPDFCERDPTVGSPGT
eas_Wnt1      GSCTVRTCWMRLPTLRAVGDVLRDRFDGASRVLYGNRGSNRASRAELLRLEPE....DPAHKP...PSPHDLVYFEKSPNFCTYSGRLGTAGT
eas_Wnt8a     GSCSIQTCWLQLADFREMGDYLKAKYDRALKIEMDKRQLRAG..NSAEGHWAP....TEAFLP...SAEAELIFLEESPDYCTHNSSLGIYGT
eas_Wnt8b     GSCTTQTCWLQLPEFREVGAHLKEKYHAALKVDLLQ...GAG..NSAAGRGAI....ADTFRS...ISTRELVHLEDSPDYCLENKTLGLLGT
eas_Wnt9a     GSCTVRTCWRQLAPFHEVGKRLKHKYETALKVGSTTNEAT....GEAGAISPPRGRAPGTGGGDPLPRTPELVHLDDSPSFCLAGRF..SPGT
eas_Wnt9b     GSCAVRTCWKQLSPFREMGQVLKLRYDSAVKVSSATNEAL....GRLELWAPARPGSPNKGLA...PRPGDLVYMEDSPSFCRPSKY..SPGT

                   300       310       320       330       340       350
eas_Wnt4      RGRTCNKTSKAID.....GCELLC..CGRGFHTAQVELAERCSCKFHWCCFVKCRQCQRLVELHTCR...................
eas_Wnt5a     QGRLCNKTSEGMD.....GCELMC..CGRGYDQFKTVQTERCHCKFHWCCYVKCKKCTEIVDQFVCK...................
eas_Wnt5b     QGRLCNKTSEGMD.....GCELMC..CGRGYDQFKSLQVERCHCKFHWCCFVKCKKCTEIVDQYVCK...................
eas_Wnt2      AGRVCNLTSRGMD.....SCEVMC..CGRGYDTSRVTRMTKCECKFHWCCAVRCQDCLEALDVHTCKAPK...SADWASPT.....
eas_Wnt2b     AGRVCSKTSKGTD.....GCEIMC..CGRGYDTTRVTRVTQCECKFHWCCAVRCKECRNTVDVHTCKAPK...KAEWLDQT.....
eas_Wnt3      RDRTCNVTSHGID.....GCDLLC..CGRGHNTRTEKRKEKCHCIFHWCCYVSCQECVRIYDVHTCK...................
eas_Wnt3a     RDRTCNVSSHGID.....GCDLLC..CGRGHNARSERRREKCHCVFHWCCYVSCQECARVYDVHTCK...................
eas_Wnt7a     QGRACNKTAPQAS.....GCDLMC..CGRGYNTHQYARVWQCNCKFHWCCYVKCNTCSERTEVYTCK...................
eas_Wnt7b     QGRLCNRTSPGAD.....GCDTMC..CGRGYNTHQYTKVWQCNCKFHWCCFVKCNTCSERTEVFTCK...................
eas_Wnt16     QGRECNRTSEGAD.....GCNLLC..CGRGYNTHVVRHVERCECKFIWCCYVRCRRCESMTDVHTCK...................
eas_Wnt11     QDRQCNKTSHGSD.....SCDLMC..CGRGYNPYTDRVVERCHCKYHWCCYVTCRRCERTVERYVCK...................
eas_Wnt6      RGRACNSSAPDLS.....GCDLLC..CGRGHRQESVQLEENCLCRFHWCCVVQCHRCRVHKELSLCL...................
eas_Wnt10a    VGRLCNKSSTGPD.....GCGSMC..CGRGHNILRQTRSERCHCRFHWCCFVVCEECRITEWVSVCK...................
eas_Wnt10b    RGRACNKTSRLLD.....GCGSLC..CGRGHNVLRQTRVERCHCRFHWCCYVLCDECKVTEWVNVCK...................
eas_Wnt1      AGRACNSSSPALD.....GCELLC..CGRGHRTRTQRVTERCT..FTERAATAQLPLRVLRE...CLSPP...GASHHLPSGFVGT
eas_Wnt8a     EGRECLQNSHNTSRWEQHSCGRLCTECGLQVEERKTEAISSCNCKFQWCCTVKCDQCRHVVNKYYCTRSP....GSAVSWGKGSAR
eas_Wnt8b     EGRECLRRGRALGRWERRSCRRLCGDCGLAVEERRAETVSSCNCKFHWCCAVRCEQCRRRVTKYFCSRAERPRGGAAHKPGRTP..
eas_Wnt9a     AGRRCHREK.........NCESIC..CGRGHNTQSRVVTRPCQCQVRWCCYVECRQCTQREEVYTCKG..................
eas_Wnt9b     AGRVCSREA.........SCSSLC..CGRGYDTQSRLVAFSCHCQVQWCCYVECQQCVQEELVYTCKH..................
```
